# A 3-D Propagation Model for Emerging Land Mobile Radio Cellular Environments

**DOI:** 10.1371/journal.pone.0132555

**Published:** 2015-08-25

**Authors:** Abrar Ahmed, Syed Junaid Nawaz, Sardar Muhammad Gulfam

**Affiliations:** Department of Electrical Engineering, COMSATS Institute of Information Technology, Islamabad, Pakistan; University of California Berkeley, UNITED STATES

## Abstract

A tunable stochastic geometry based Three-Dimensional (3-D) scattering model for emerging land mobile radio cellular systems is proposed. Uniformly distributed scattering objects are assumed around the Mobile Station (MS) bounded within an ellipsoidal shaped Scattering Region (SR) hollowed with an elliptically-cylindric scattering free region in immediate vicinity of MS. To ensure the degree of expected accuracy, the proposed model is designed to be tunable (as required) with nine degrees of freedom, unlike its counterparts in the existing literature. The outer and inner boundaries of SR are designed as independently scalable along all the axes and rotatable in horizontal plane around their origin centered at MS. The elevated Base Station (BS) is considered outside the SR at a certain adjustable distance and height w.r.t. position of MS. Closed-form analytical expressions for joint and marginal Probability Density Functions (PDFs) of Angle-of-Arrival (AoA) and Time-of-Arrival (ToA) are derived for both up- and down-links. The obtained analytical results for angular and temporal statistics of the channel are presented along with a thorough analysis. The impact of various physical model parameters on angular and temporal characteristics of the channel is presented, which reveals the comprehensive insight on the proposed results. To evaluate the robustness of the proposed analytical model, a comparison with experimental datasets and simulation results is also presented. The obtained analytical results for PDF of AoA observed at BS are seen to fit a vast range of empirical datasets in the literature taken for various outdoor propagation environments. In order to establish the validity of the obtained analytical results for spatial and temporal characteristics of the channel, a comparison of the proposed analytical results with the simulation results is shown, which illustrates a good fit for 10^7^ scattering points. Moreover, the proposed model is shown to degenerate to various notable geometric models in the literature by an appropriate choice of a few parameters.

## 1 Introduction

Internet Protocol (IP) based network architecture in 4th Generation (4G) wireless communications promise a data rate up to 1Gbps at low mobility and 100 Mbps at high mobility [1]. However, still there is a drastic increase in the data traffic and capacity demands due to an increase in the data subscribers and bandwidth-intensive applications. Along with various other dynamic demands, the demands of low latency for real-time applications, dynamic framework, and efficient energy management [2] have triggered the investigation of 5th Generation (5G) for next generation communication networks. Advanced spatial filtering techniques implemented at the base station (BS) are among the potential candidates to significantly enhance the capacity of future land mobile radio cellular systems [1, 3, 4]. In emerging cellular communication systems, the less elevated BSs and small sized cells lead to an equal importance of dispersion of multipath waves in the elevation plane as compared to the azimuth plane [4]. Performance of antenna array systems and signal processing techniques implemented at the BS strongly depends on the available knowledge of the radio channel’s characteristics regarding the dispersion of multipath waves in horizontal and vertical planes. Accurate knowledge of radio channel characteristics is of immense importance to meet extremely challenging requirements of the emerging future generation communication networks. The existing widely used radio channel models (e.g., COST 2100, WINNER, and ITU IMT Advanced) are not adequate for the 5G potential candidate technologies because of numerous strong and obvious reasons including the radically higher frequency range (Millimetre wave) [5], significantly larger antenna arrays with higher directional resolution (massive MIMO) [3], dual end mobility of communication nodes (Vehicle to Vehicle) [6, 7], considerably smaller and denser cells [4], and substantially less elevated BSs [1, 4]. In recent years, extensive research is being conducted for the adaption of such dynamic aspects of 5G networks by precise modelling of the propagation channel’s behavior. Therefore, various stochastic geometry-based three-dimensional (3-D) models for land mobile radio cellular systems are presented in the literature [8–17], which integrate dispersion of multipath waves in elevation plane for characterization of the terrestrial radio cellular channels. Despite these developments, there still is a substantial scope to extend the research work for development of realistic three dimensional not oversimplified channel models which are tunable to adapt various emerging communication scenarios.

The waves scattered from the high-rise structures do not propagate horizontally, which correspond in an elevational angular spread of up to an angle of 20° [9, 14]. A 3-D semi-spheroid model for macrocellular channels is proposed in [9], where spatial characteristics of the channel observed at both ends of the communication link are analysed. However, this model does not provide an analysis on temporal characteristics of the channel. In [10], closed form expressions for joint distribution of Time-of-Arrival (ToA) and 2-D Angle-of-Arrival (AoA) are derived observed from both ends of the link. Whereas, this model considers both ends of the communication link at an equal elevation, which limits the usability of the channel model. Another 3-D model for macro-cellular environment is proposed in [11], which considers the elevated BS equipped with a directional antenna. The impact of beam-width of directional antenna at spatial and temporal characteristics of the channel is intensively analysed. The antenna employed at the elevated BS is considered directional with a scalable beam-width only in the azimuth plane, with an omnidirectional radiation in elevation plane, which may not be a reasonable assumption for future less elevated BS and small sized cells scenarios. The channel model in [11] is further extended in [12], for classification of time varying nature of the channel. In [13], a geometry-based 3-D model for indoor radio propagation environments is presented and spatio-temporal characteristics of the channel are discussed. In [14], a 3-D model with a 2-D disk shaped SR around mobile station (MS) is presented with the BS considered elevated outside the SR. The obtained analytical results for angular characteristics of the channel are also compared with simulation results and empirical datasets. Expressions for the probability density function (PDF) of the elevation angle to the power spectral density of the signals received at BS in the 3-D model have been derived in [15], and functions for PDF of the elevation angle have been proposed. A geometry-based 3-D channel model for spatio-temporal analysis of emerging vehicle-to-vehicle (V2V) communication links is presented in [16]. The model in [16] is further extended in [18] by introducing another degree of freedom which allows flexibility in scattering region to introduce a hollow scattering free region in close vicinity of mobile nodes. In [17], scatterers are assumed to be uniformly distributed within the volume of a 3-D cylinder above the ground plane and mathematical expressions for tri- and bi-variate ToA and 2-D AoA of the uplink have been derived. The 3-D models discussed above, consider the scattering objects as uniformly distributed confined within a certain geometric region. A Gaussian distribution of scattering objects based 3-D model for macrocell environment is proposed in [19], and its characterization for spatio-temporal statistics is presented. In [20], a cylindric region with flat bottom above the ground plane is considered to model the scattering objects and the density of scatterers is modeled to reduce (sparse) along the elevation axis and AoA characteristics of the uplink are discussed.

In various realistic outdoor propagation scenarios, the effective multipath waves corresponded from the distant scattering objects and the close vicinity of MS is usually scattering free region (e.g., less populated streets, urban areas). For such communication scenarios, a number of stochastic geometry-based channel models are proposed in the literature [21–25], which assume the local vicinity of MS as scattering free region. A hollow-disc of uniformly distributed scattering points is considered in [21], for modeling the SR. Closed-form expressions for the joint and marginal PDFs of ToA and AoA of uplink/downlink have been derived. In [22], a hollow circular (annular strips) shaped SR based channel model for V2V communications has been proposed and detailed analyses for angular and temporal statistics of the channel are presented. In [23], uniformly distributed scatterers are considered confined in hollow elliptical shaped SRs around both ends of the communication link. Closed-form expressions for the PDF of azimuth AoA of incoming multipaths at each unit are derived and the impact of scatterer’s distribution on the angular spread of the received signal is presented. In [24], scatterers are modeled as confined within an elliptical space with a hollow disk and spatial temporal statistics of the channel are discussed. In [25] scatterers are assumed to be present in uniformly shaped spatial density in a hollow disc centered around MS, the obtained AoA and ToA characteristics are compared with customary results for both indoor and outdoor environments. There is no such model in the literature (within the scope of the authors’ knowledge), which integrates both the azimuth and elevation planes (i.e., 3-D SR) for the propagation of multipath waves and provides tunable SR (independently scalable and rotatable along all axes) to model various practical propagation scenarios, like, different street orientations, high rise structures, and scattering free regions in the close vicinity of communicating nodes, etc.

In this paper, a stochastic geometry-based 3-D scattering model is proposed which considers uniformly distributed scattering objects confined within a rotatable ellipsoidal region hollowed with a rotatable elliptical-cylindric region around the MS. The outer bounding ellipsoid and inner bounding elliptical-cylinder are designed as independently scalable along all axes and independently rotatable around the MS on the horizontal plane. Analytical expressions for joint and marginal PDF of AoA in azimuth and elevation planes are derived. The rest of the paper is organized as follows: definitions of the model’s parameters is given in Section 2. The proposed 3-D scattering model is presented in Section 3. In Section 4, joint and marginal PDF of AoA for azimuth and elevation angles seen at BS and MS are derived. Joint and marginal PDF of ToA for azimuth and elevation angles seen at BS and MS are derived in Section 5. The effect of various physical model parameters on the obtained analytical results for azimuth and elevation AoA and validation of the proposed model are presented in Section 6. Directions for extension of the proposed work are given in Section 7. Finally the paper is concluded in Section 8.

## 2 Nomenclature

Definition of the proposed model’s parameters is given in Table 1.

**Table 1:** Definition of Symbols.

Symbol	Definition
*h*_*b*_	Height of BS.
*d*	Horizontal distance of MS from BS.
*a*_*o*_, *b*_*o*_, and *c*_*o*_	Major, intermediate and minor axes of the outer bounding ellipsoid.
*a*_*i*_ and *b*_*i*_	Major and minor axes of the inner bounding elliptical-cylinder.
*θ*_*o*_ and *θ*_*i*_	Rotation angle of the outer bounding ellipsoid and the inner bounding elliptical-cylinder.
*s*_*p*_	*p* ^*th*^ scattering point.
*ϕ*_*m*_ and *β*_*m*_	Azimuth and elevation angles observing from MS.
*ϕ*_*b*_ and *β*_*b*_	Azimuth and elevation angles observing from BS.
*V*_*i*_	Volume of the effective SR.
$\rho_{\phi \text{o}}^{+}$ and $\rho_{\phi \text{i}}^{+}$	Maximum horizontal distance of the the outer bounding ellipsoid and the inner bounding elliptical-cylinder from BS.
$\rho_{\phi \text{o}}^{-}$ and $\rho_{\phi \text{i}}^{-}$	Minimum horizontal distance of the the outer bounding ellipsoid and the inner bounding elliptical-cylinder from BS.
$\rho_{\beta \text{o}}^{+}$ and $\rho_{\beta \text{i}}^{+}$	Maximum line of sight distance of the the outer bounding ellipsoid and the inner bounding elliptical-cylinder from BS.
$\rho_{\beta \text{o}}^{-}$ and $\rho_{\beta \text{i}}^{-}$	Minimum line of sight distance of the the outer bounding ellipsoid and the inner bounding elliptical-cylinder from BS.
*ρ*_*g*_	The distance from BS to the ground plane (for a given azimuth and elevation angle).
*β*_min_ and *β*_max_	Minimum and maximum elevation angles.
*β*_*t*_	Threshold elevation angles for different partitions of SR.
*r*_*o*_ and *r*_*i*_	Distance of MS from farthest and nearest scatterer (for a given azimuth and elevation angle).
*h*_*e*,*b*_ and *h*_*e*,*m*_	Height of cylinder observing from BS and MS (for a given azimuth and elevation angle).
*ϕ*_*t*o_ and *ϕ*_*t*i_	Azimuth threshold angles of outer bounding ellipsoid and the inner bounding elliptical-cylinder observing from BS.
*τ*_*o*_ and *τ*_max_	Delay of line of sight and longest path (Minimum and maximum delay).
*τ*_*m*,min_ and *τ*_*m*,max_	Delay of shortest and longest path observing from MS (for a given azimuth and elevation angle).
*τ*_*b*,min_ and *τ*_*b*,max_	Delay of shortest and longest path observing from BS (for a given azimuth and elevation angle).
*r*_*m*_ and *r*_*b*_	Distance of a scatterer from MS and BS.

## 3 System Model

**Fig 1:**
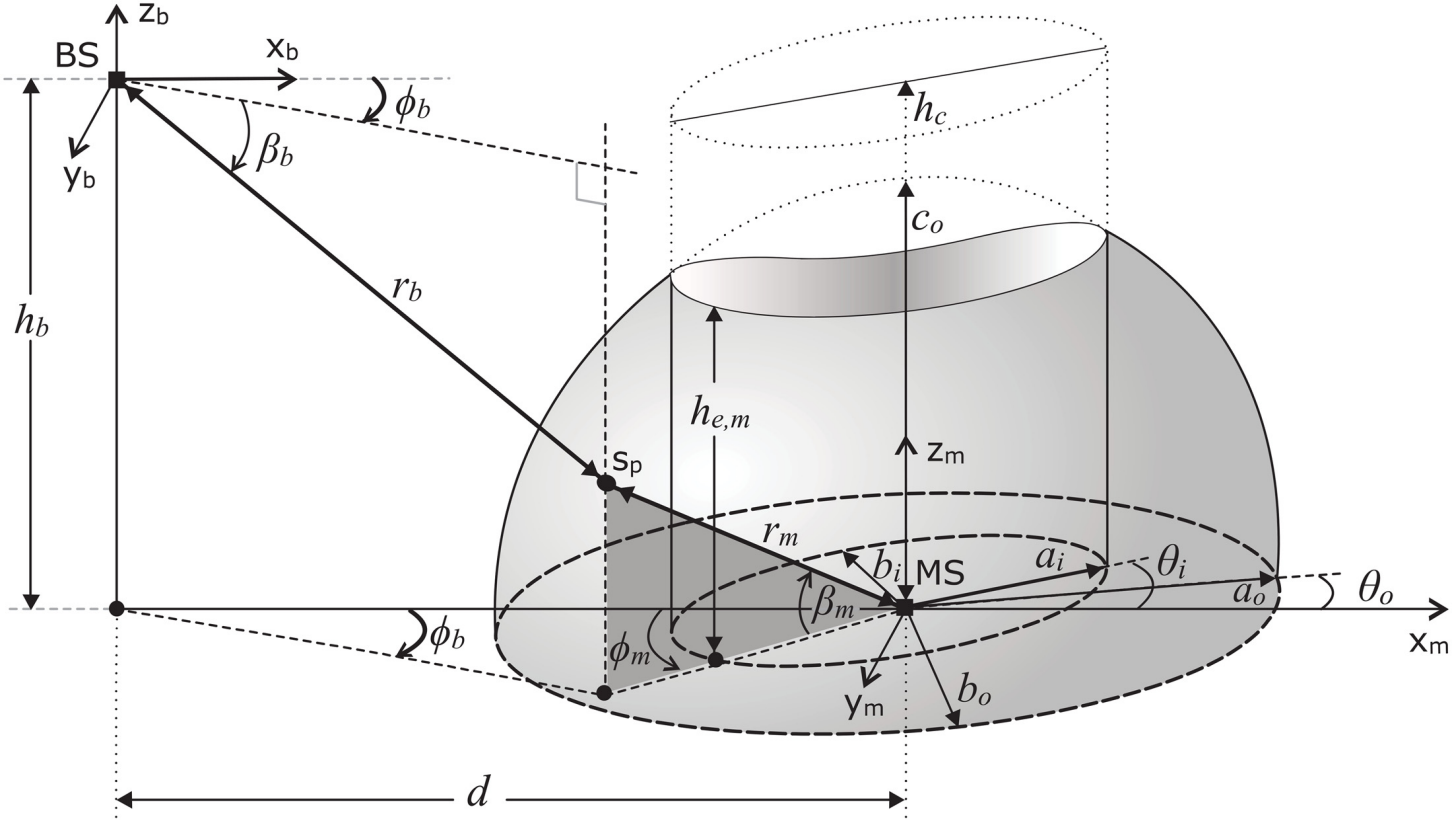
Proposed 3-D scattering model for outdoor radio cellular propagation environments.

In this section, the proposed 3-D ellipsoidal model is presented. Uniformly distributed scattering objects around the MS are bounded in an ellipsoid and elliptical-cylinder, as shown in Fig 1. The BS is assumed at an height *h*_*b*_ and at a distance *d* from the MS. The outer bounding ellipsoid is designed as independently scalable along its major, intermediate, and minor axes with parameters *a*_*o*_, *b*_*o*_, and *c*_*o*_, respectively. In outdoor radio propagation environments, the multipath waves from the elevation plane do not often arrive at MS from the directions exactly perpendicular to the ground plane. To ensure appropriate modeling of SR, the immediately adjacent region around MS is designed as scattering free by modeling the geometry of this hollow region with an adjustable elliptical-cylinder. Height of the cylinder *h*_*c*_ is fixed to be greater than *c*_*o*_, such that it eliminates all the scattering objects elevated exactly above the MS. The spread of multipath waves in elevation plane depends on the vertical structures in the vicinity of MS; therefore, to adapt a certain propagation environment, the inner bounding elliptical-cylinder is modeled as scalable along its major and minor axes with parameters *a*_*i*_ and *b*_*i*_, respectively. Both the outer and inner bounding geometric shapes of SR are designed as rotatable around MS in x-y plane (with parameters, *θ*_*o*_ and *θ*_*i*_) to appropriately model various orientations of effective SR (e.g., modeling street orientations). Therefore, the proposed model provides nine degrees of freedom (i.e., *a*_*o*_, *b*_*o*_, *c*_*o*_, *θ*_*o*_, *a*_*i*_, *b*_*i*_, *θ*_*i*_, *d*, and *h*_*b*_) to ensure the degree of expected accuracy. The linear distance of a certain scattering point (*s*_*p*_) from MS and BS is shown by *r*_*m*_ and *r*_*b*_, respectively. The azimuth and elevation angle of arriving multipath wave at the BS corresponded from a certain scattering point is represented by *ϕ*_*b*_ and *β*_*b*_, respectively. The azimuth and elevation angles seen at MS are shown by *ϕ*_*m*_ and *β*_*m*_, respectively. Some common assumptions (as in [9–11]) taken to design the proposed model are given below,

1. The SR is defined by the non-overlapping portion of the outer-bounding ellipse and inner-hollow cylinder.
2. Scatterers are assumed uniformly distributed in the vicinity of MS confined within the defined hollow semi-ellipsoid SR.
3. The signal arriving at a scattering object is assumed to be scattered with an equal power in all the directions(omnidirectional lossless re-radiation) in 3-D space.
4. All the multipath components received at MS are assumed to have equal scattering coefficients and uniform random phases.
5. The communication is assumed to take place via single bounce from an isotropic scattering object.
6. The location of any scattering point (*s*_*p*_) is represented in the Cartesian coordinate system by (*x*_*s*_*p*__, *y*_*s*_*p*__, *z*_*s*_*p*__) or in spherical coordinate system by (*r*_*s*_*p*__, *ϕ*_*s*_*p*__, *β*_*s*_*p*__).

The outer bounding ellipse can be defined as,

$$\frac{{\left(x_{o}\cos\theta_{o}+y_{o}\sin\theta_{o}\right)}^{2}}{a_{o}^{2}}+\frac{{\left(-x_{o}\sin\theta_{o}+y_{o}\cos\theta_{o}\right)}^{2}}{b_{o}^{2}}+\frac{z_{o}^{2}}{c_{o}^{2}}=1,$$ (1)

where, *x*_*o*_ = *r*_*o*_ cos *β*_*m*_ cos *ϕ*_*m*_, *y*_*o*_ = *r*_*o*_ cos *β*_*m*_ sin *ϕ*_*m*_, and *z*_*o*_ = *r*_*o*_ sin *β*_*m*_ represent the coordinates of outer bounding ellipsoid. Seeing from MS with a given azimuth angle *ϕ*_*m*_ and at given elevation angle *β*_*m*_, the distance from MS to the nearest and farthest scatterer is *r*_*i*_ and *r*_*o*_, respectively, given by

$$r_{i}=\frac{1}{\cos\beta_{m}}\sqrt{\frac{2(a_{i}^{2}b_{i}^{2})}{a_{i}^{2}+b_{i}^{2}+\left(b_{i}^{2}-a_{i}^{2}\right)\cos2\left(\theta_{i}-\phi_{m}\right)}},$$ (2)

$$r_{o}=\frac{a_{o}b_{o}c_{o}}{\sqrt{a_{o}^{2}b_{o}^{2}\sin^{2}\beta_{m}+c_{o}^{2}\cos^{2}\beta_{m}\left(b_{o}^{2}\cos^{2}\left(\theta_{o}-\phi_{m}\right)+a_{o}^{2}\sin^{2}\left(\theta_{o}-\phi_{m}\right)\right)}}$$ (3)

Volume (*V*_*i*_) of the effective illuminated SR can be found by subtracting the volume of hollow elliptical-cylinder region (*V*_*c*_) from the volume of semi ellipsoid (*V*_*e*_), i.e., *V*_*i*_ = *V*_*e*_ − *V*_*c*_; which can be expressed as,

$$V_{i}=\frac{2}{3}\pi a_{o}b_{o}c_{o}-2\int_{0}^{\gamma_{m}}\frac{c_{\gamma o}}{a_{\gamma o}}\int_{\eta_{l}}^{\eta_{u}}\sqrt{{a_{\gamma o}}^{2}-\eta^{2}}\;d\eta \;d\gamma ,$$ (4)

where, *γ* represents the y-coordinate of an arbitrary vertical plane parallel to x-z plane and *η* represents the x-coordinate of an arbitrary horizontal plan parallel to y-z plane.

$$\begin{array}{c} V_{i}=\frac{2}{3}\pi a_{o}b_{o}c_{o}-2\int_{0}^{\gamma_{m}}\frac{c_{\gamma o}}{2a_{\gamma o}}\{\eta_{u}\sqrt{{a_{\gamma o}}^{2}-\eta_{u}^{2}}-\eta_{l}\sqrt{{a_{\gamma o}}^{2}-\eta_{l}^{2}}\\ \;+{a_{\gamma o}}^{2}\arctan(\frac{\eta_{u}}{\sqrt{{a_{\gamma o}}^{2}-\eta_{u}^{2}}})-{a_{\gamma o}}^{2}\arctan(\frac{\eta_{l}}{\sqrt{{a_{\gamma o}}^{2}-\eta_{l}^{2}}})\}d\gamma , \end{array}$$ (5)

where, *a*_*γo*_, *c*_*γo*_, *η*_*u*_, *η*_*l*_, and *γ*_*m*_ can be found as,

$$a_{\gamma o}=\frac{\sqrt{2a_{o}^{2}b_{o}^{2}\left(a_{o}^{2}+b_{o}^{2}-2\gamma^{2}+\left(b_{o}^{2}-a_{o}^{2}\right)\cos\left(2\theta_{o}\right)\right)}}{a_{o}^{2}+b_{o}^{2}+\left(b_{o}^{2}-a_{o}^{2}\right)\cos\left(2\theta_{o}\right)},$$ (6)

$$c_{\gamma o}=\sqrt{-\frac{c_{o}^{2}\left(2\gamma^{2}-a_{o}^{2}-b_{o}^{2}+\left(a_{o}^{2}-b_{o}^{2}\right)\cos\left(2\theta_{o}\right)\right)}{a_{o}^{2}+b_{o}^{2}+\left(b_{o}^{2}-a_{o}^{2}\right)\cos\left(2\theta_{o}\right)}},$$ (7)

$$\begin{array}{c} \begin{array}{c} \eta_{u}\\ \eta_{l} \end{array}\}=\pm \{\sqrt{-2a_{i}^{2}b_{i}^{2}\left(2\gamma^{2}-a_{i}^{2}-b_{i}^{2}+\left(a_{i}^{2}-b_{i}^{2}\right)\cos\left(2\theta_{i}\right)\right)}\pm \left(a_{i}^{2}-b_{i}^{2}\right)\gamma \sin\left(2\theta_{i}\right)\}\\ \;\;\;\times \frac{1}{a_{i}^{2}+b_{i}^{2}+\left(b_{i}^{2}-a_{i}^{2}\right)\cos\left(2\theta_{i}\right)}\pm a_{\gamma o}-\frac{1}{a_{o}^{2}+b_{o}^{2}+\left(b_{o}^{2}-a_{o}^{2}\right)\cos\left(2\theta_{o}\right)}\\ \;\;\;\times \{\left(a_{o}^{2}-b_{o}^{2}\right)\gamma \sin\left(2\theta_{o}\right)\pm \sqrt{-2a_{o}^{2}b_{o}^{2}\left(2\gamma^{2}-a_{o}^{2}-b_{o}^{2}+\left(a_{o}^{2}-b_{o}^{2}\right)\cos\left(2\theta_{o}\right)\right)}\}, \end{array}$$ (8)

$$\gamma_{m}=\cos\theta_{i}\sqrt{b_{i}^{2}+a_{i}^{2}\tan^{2}\theta_{i}}.$$ (9)

**Fig 2:**
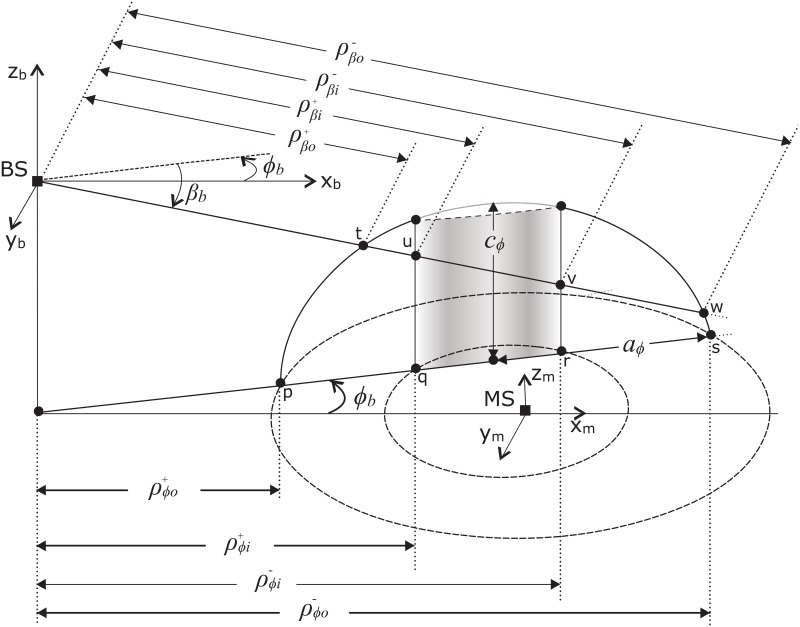
Cross section view of proposed SR for a certain direction of observation from BS.

A horizontal line drawn from the base of BS at a given azimuth angle *ϕ*_*b*_, intersects the outer bounding ellipsoid at points p and s, and hollow elliptical-cylinder at points q and r as shown in Fig 2. After doing tedious mathematical simplifications, distance from BS to these points can be obtained as given below,

$$\begin{array}{c} \begin{array}{c} \rho_{\phi k}^{+}\\ \rho_{\phi k}^{-} \end{array}\}=-\{2d\left(b_{k}^{2}\cos\theta_{k}\cos\left(\theta_{k}-\phi_{b}\right)+a_{k}^{2}\sin\theta_{k}\sin\left(\theta_{k}-\phi_{b}\right)\right)\pm (2a_{k}^{2}b_{k}^{2}\cos^{2}\phi_{b}(a_{k}^{2}+b_{k}^{2}\\ -d^{2}+\left(b_{k}^{2}-a_{k}^{2}\right)\cos\left(2\left(\theta_{k}-\phi_{b}\right)\right)+d^{2}\cos\left(2\phi_{b}\right){))}^{\frac{1}{2}}\}\frac{1}{a_{k}^{2}+b_{k}^{2}+\left(b_{k}^{2}-a_{k}^{2}\right)\cos\left(2\left(\theta_{k}-\phi_{b}\right)\right)}. \end{array}$$ (10)

The horizontal distances from BS to the intersection points for outer bounding ellipsoid and inner cylinder can be obtained by setting k = *o* and k = *i* in Eq (10) for $\rho_{\phi o}^{\pm }$ and $\rho_{\phi i}^{\pm }$, respectively. A vertical 2-D elliptical shaped region is formed for the given observing azimuth AoA *ϕ*_*b*_. A line drawn from BS for a given elevation angle *β*_*b*_ intersects this 2-D vertical ellipse and hollow cylinder at points t and w and at u and v, respectively (see Fig 2). The major and minor axes *a*_*ϕ*_ and *c*_*ϕ*_ of the aforementioned 2-D vertical ellipse are given as follows,

$$\begin{array}{c} a_{\phi }=\frac{1}{\left(a_{o}^{2}+b_{o}^{2}+\left(b_{o}^{2}-a_{o}^{2}\right)\cos2\left(\theta_{o}-\phi_{b}\right)\right)\cos\phi_{b}}\\ \;\;\;\times {\left\{-2a_{o}^{2}b_{o}^{2}\cos^{2}\phi_{b}\left(d^{2}-a_{o}^{2}-b_{o}^{2}+\left(a_{o}^{2}-b_{o}^{2}\right)\cos2\left(\theta_{o}-\phi_{b}\right)-d^{2}\cos2\phi_{b}\right)\right\}}^{\frac{1}{2}}, \end{array}$$ (11)

$$c_{\phi }=-\frac{c_{o}}{a_{o}b_{o}}\sqrt{a_{o}^{2}b_{o}^{2}-a_{o}^{2}x_{e}^{2}\sin^{2}\theta_{e}-b_{o}^{2}x_{e}^{2}\cos^{2}\theta_{e}},$$ (12)

where, the parameters *x*_*e*_ and *θ*_*e*_ are given as under,

$$x_{e}=\sqrt{d^{2}+{\left(a_{\phi }+\rho_{\phi o}^{+}\right)}^{2}-2d\left(a_{\phi }+\rho_{\phi o}^{+}\right)\cos\phi_{b}},$$ (13)

$$\theta_{e}=\{\begin{array}{cc} \pi -(\theta_{r}+\theta_{o}) & ;\;\phi_{b}<0\\ \theta_{r}+\theta_{o} & ;\;\text{otherwise} \end{array},$$ (14)

where, *θ*_*r*_ can be written as,

$$\theta_{r}=\{\begin{array}{cc} \arcsin\frac{\left(a_{\phi }+\rho_{\phi o}^{+}\right)\sin\phi_{b}}{xe} & ;\;\phi_{b}\neq 0\\ 0 & ;\;\phi_{b}=0 \end{array}.$$ (15)

Observing from the BS (i.e., given *ϕ*_*b*_ and *β*_*b*_) the distances to the intersection points (t,w and u,v) are shown as $\rho_{\beta o}^{\pm }$ and $\rho_{\beta i}^{\pm }$, which can be found by the expressions given below,

$$\begin{array}{c} \begin{array}{c} \rho_{\beta o}^{+}\\ \rho_{\beta o}^{-} \end{array}\}=\sec\beta_{b}\{\frac{1}{a_{\phi }^{2}+c_{\phi }^{2}+\left(c_{\phi }^{2}-a_{\phi }^{2}\right)\cos\left(2\beta_{b}\right)}(\left(a_{\phi }+\rho_{\phi o}^{+}\right)\\ \;\;-(2a_{\phi }^{2}\sin\beta_{b}\left(\left(a_{\phi }+\rho_{\phi o}^{+}\right)\sin\beta_{b}-h_{b}\cos\beta_{b}\right)\pm (2(a_{\phi }^{2}c_{\phi }^{2}\cos^{2}\beta_{b}(a_{\phi }^{2}+c_{\phi }^{2}-{\left(a_{\phi }+\rho_{\phi o}^{+}\right)}^{2}\\ \;\;-h_{b}^{2}+\left(c_{\phi }^{2}-a_{\phi }^{2}+{\left(a_{\phi }+\rho_{\phi o}^{+}\right)}^{2}-h_{b}^{2}\right)\cos\left(2\beta_{b}\right)+2dh_{b}\sin\left(2\beta_{b}\right){)))}^{\frac{1}{2}}))\}, \end{array}$$ (16)

$$\rho_{\beta i}^{\pm }=\rho_{\phi i}^{\pm }\sec\beta_{b}.$$ (17)

For a given direction of observation (i.e., given *β*_*b*_ and *ϕ*_*b*_), the distance from BS to the ground plane is given by *ρ*_*g*_,

$$\rho_{g}=\frac{h_{b}}{\sin\beta_{b}}.$$ (18)

**Fig 3:**
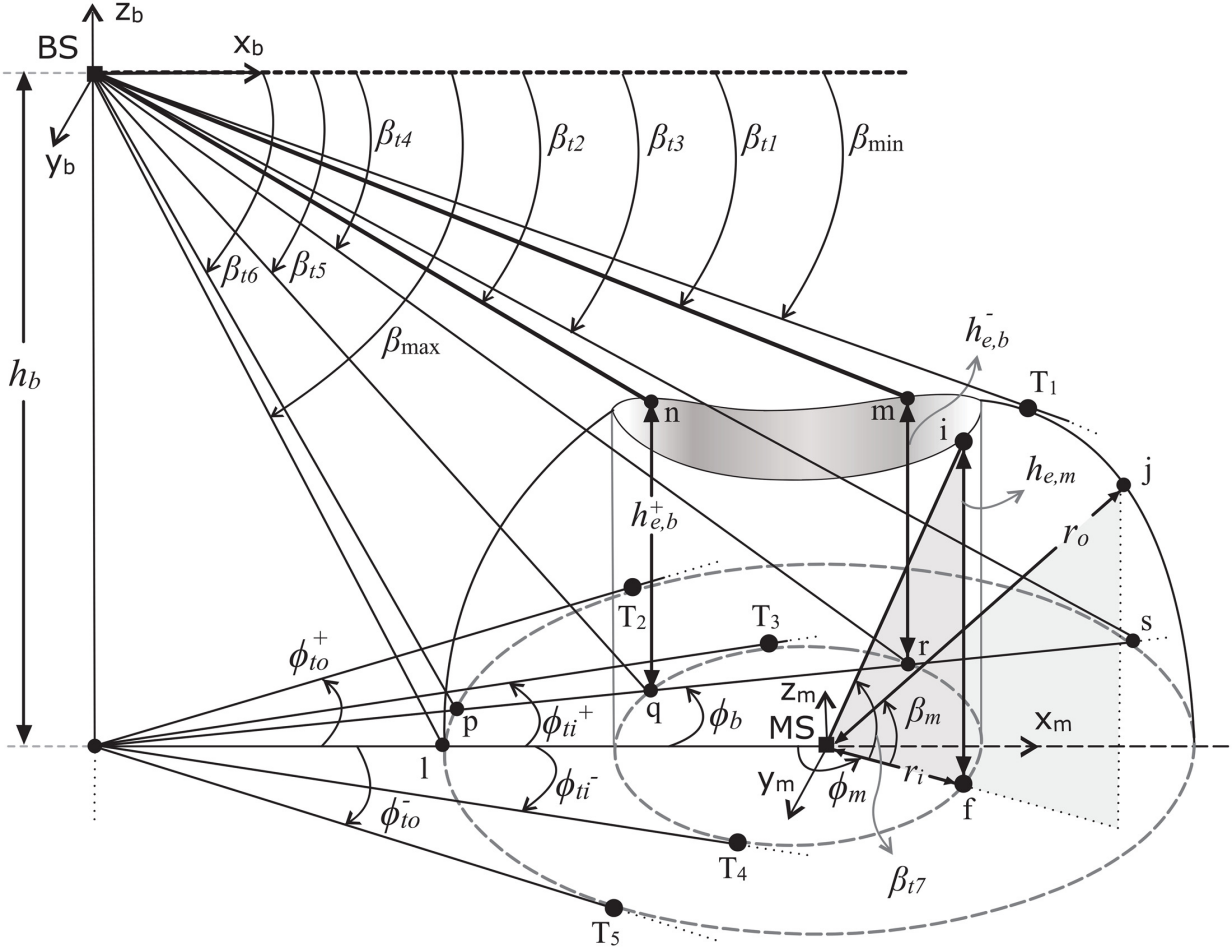
Elevation and azimuth threshold angles to define angular partitions of SR.

Proposed scattering model with elevation and azimuth threshold angles is shown in Fig 3, where the elevation and azimuth threshold angles are shown by *β*_*t*_ and *ϕ*_*t*_, respectively. The angles *β*_min_ and *β*_max_ are calculated to define the limits of angular spread in elevation plane observed at BS and are given below,

$$\beta_{\text{min}}=\arctan(\frac{h_{b}d-\sqrt{h_{b}^{2}a_{o}^{2}+c_{o}^{2}\left(d^{2}-a_{o}^{2}\right)}}{d^{2}-a_{o}^{2}}),$$ (19)

$$\beta_{\text{max}}=\arctan(\frac{h_{b}}{d-a_{o}}).$$ (20)

The elevation threshold angles *β*_t1_ to *β*_t6_ are important angles based on geometrical composition of SR. Angles formed by the lines from BS which intersect the top of inner cylinder at points m and n, shown as *β*_t1_ and *β*_t2_, are given below,

$$\begin{array}{c} \begin{array}{c} \beta_{t1}^{-}\\ \beta_{t2}^{+} \end{array}\}=\arctan(\frac{h_{b}+h_{e,b}^{\mp }}{\rho_{\phi i}^{\mp }}), \end{array}$$ (21)

where, the height $h_{e,b}^{\pm }$ of cylinder for given *ϕ*_*b*_ and *β*_*b*_ can be found as,

$$h_{e,b}^{\pm }=\frac{c_{o}}{a_{o}b_{o}}{\left\{\frac{1}{2}(a_{o}^{2}\left(2b_{o}^{2}-d^{2}-\rho_{\phi i}^{\pm }{}^{2}\right)-b_{o}^{2}\left(d^{2}+\rho_{\phi i}^{\pm }{}^{2}\right)\;+\left(a_{o}^{2}-b_{o}^{2}\right)\text{cos}\left(2\left(\alpha -\theta_{o}\right)\right)\left(d^{2}+\rho_{\phi i}^{\pm }{}^{2}-2d\rho_{\phi i}^{\pm }\text{cos}\;\phi_{b}\right))\right\}}^{\frac{1}{2}}.$$ (22)

The simplification parameter *α* is given by,

$$\alpha =\pi -\arcsin(\frac{\rho_{\phi i}^{\pm }\sin\phi_{b}}{\sqrt{d^{2}+{\rho_{\phi i}^{\pm }}^{2}-2d\rho \cos\phi_{b}}}).$$ (23)

The lines drawn from BS at a given azimuth angle to the base of inner cylinder form the angles *β*_t4_ and *β*_t5_, respectively, as shown below,

$$\begin{array}{c} \begin{array}{c} \beta_{t4}^{-}\\ \beta_{t5}^{+} \end{array}\}=\arctan(\frac{h_{b}}{\rho_{\phi i}^{\mp }}). \end{array}$$ (24)

For a given azimuth angle, the elevation angles formed with the base of outer bounding ellipse are shown by *β*_t3_ and *β*_t6_, which can be found as,

$$\begin{array}{c} \begin{array}{c} \beta_{t3}^{-}\\ \beta_{t6}^{+} \end{array}\}=\arctan(\frac{h_{b}}{\rho_{\phi o}^{\mp }}). \end{array}$$ (25)

The azimuth threshold angles which separate the angular partitions of SR can be found as,

$$\begin{array}{c} \phi_{tk}^{\pm }=\arctan\{\frac{1}{a_{k}^{2}\cos^{2}\theta_{k}+b_{m}^{2}\sin^{2}\theta_{k}-d^{2}}\left(a_{k}^{2}-b_{k}^{2}\right)\cos\theta_{k}\sin\theta_{k}\\ \;\;\;\pm {\left\{d^{2}\left(a_{k}^{2}\sin^{2}\theta_{k}+b_{k}^{2}\cos^{2}\theta_{k}\right)-a_{k}^{2}b_{k}^{2}\right\}}^{\frac{1}{2}}\}. \end{array}$$ (26)

The azimuth threshold angles formed with horizontal lines drawn from BS to the tangent points at outer (T_2_ and T_5_) and inner (T_3_ and T_4_) bounding elliptical regions, can be obtained by setting k = *o* and k = *i* in Eq (26) for $\phi_{t\text{o}}^{\pm }$ and $\phi_{t\text{i}}^{\pm }$, respectively. When observing from MS, at a certain azimuthal and elevation angle, the height of hollow cylindric region *h*_*e*,*m*_ and its subtended angle *β*_t7_ can be expressed as,

$$h_{e,m}=c_{o}\sqrt{\frac{4a_{o}^{4}b_{o}^{4}-r_{i}^{2}\cos^{2}\beta_{m}{\left(a_{o}^{2}+b_{o}^{2}+\left(-a_{o}^{2}+b_{o}^{2}\right)\cos2\left(\theta_{o}-\phi_{m}\right)\right)}^{2}}{2a_{o}^{2}b_{o}^{2}\left(a_{o}^{2}+b_{o}^{2}+\left(-a_{o}^{2}+b_{o}^{2}\right)\cos2\left(\theta_{o}-\phi_{m}\right)\right)}},$$ (27)

$$\beta_{t7}=\arctan(\frac{h_{e,m}}{r_{i}\cos\beta_{m}}).$$ (28)

The transformation relations between cartesian and spherical coordinate systems are *x*_*m*_ = *r*_*m*_ cos *β*_*m*_ cos *ϕ*_*m*_, *y*_*m*_ = *r*_*m*_ cos *β*_*m*_ sin *ϕ*_*m*_, and *z*_*m*_ = *r*_*m*_ sin *β*_*m*_.

## 4 Spatial characteristics of the radio channel

In this section, we derive the joint and marginal PDFs of AoA seen at MS and BS in subsections 4.1 and 4.2, respectively.

### 4.1 PDF of AoA at MS

The joint density function of AoA seen at MS and radius *r*_*m*_ can be found as,

$$p\left(r_{m},\phi_{m},\beta_{m}\right)=\frac{f(x_{m},y_{m},z_{m})}{\left|J(x_{m},y_{m},z_{m})\right|}.$$ (29)

The Jacobian transformation *J*(*x*_*m*_, *y*_*m*_, *z*_*m*_) of Eq (29) can be found as shown below,

$$\begin{array}{c} J\left(x_{m},y_{m},z_{m}\right)=\;{\left|\begin{array}{ccc} \cos\beta_{m}\sin\phi_{m} & r_{m}\cos\beta_{m}\cos\phi_{m} & -r_{m}\sin\beta_{m}\sin\phi_{m}\\ \cos\beta_{m}\cos\phi_{m} & -r_{m}\cos\beta_{m}\sin\phi_{m} & -r_{m}\sin\beta_{m}\cos\phi_{m}\\ \sin\beta_{m} & 0 & r_{m}\cos\beta_{m} \end{array}\right|}^{-1}. \end{array}$$ (30)

As the scatterers are assumed to be uniformly distributed in the SR with volume *V*_*i*_, the scatterer density function can thus be found as,

$$f\left(x_{m},y_{m},z_{m}\right)=\{\begin{array}{cc} \frac{1}{V_{i}} & ;\;(x_{m},y_{m},z_{m})\in \text{SR}\\ 0 & ;\;\text{otherwise} \end{array}$$ (31)

By substituting Eqs (30) and (31) in Eq (29), the joint density function can be written as,

$$p\left(r_{m},\phi_{m},\beta_{m}\right)=\frac{r_{m}^{2}\cos\beta_{m}}{V_{i}}.$$ (32)

Integrating the joint density function given in Eq (32) over *r*_*m*_ ranging from *r*_*i*_ to *r*_*o*_,

$$p\left(\phi_{m},\beta_{m}\right)=\frac{{\left(r_{o}-r_{i}\right)}^{3}\cos\beta_{m}}{3V_{i}},$$ (33)

where, (*r*_*o*_ − *r*_*i*_) is the effective amount of scattering objects. The marginal PDF of azimuth AoA seen at MS can thus be obtained by integrating Eq (33) over *β*_*m*_ for its appropriate limits as under,

$$\begin{array}{c} p\left(\phi_{m}\right)=\int_{0}^{\beta_{t7}}p\left(\phi_{m},\beta_{m}\right)d\beta_{m}. \end{array}$$ (34)

The closed-form solution of above equation can thus be expressed in simplified form as under,

$$\begin{array}{c} p\left(\phi_{m}\right)=\frac{2\sqrt{2}}{3V_{i}\kappa_{3}\sqrt{\kappa_{4}}}\{\kappa_{1}^{3}\kappa_{3}\sqrt{\kappa_{4}}\;\tan\beta_{t7}+3\kappa_{1}^{2}\sqrt{\kappa_{2}}\kappa_{3}^{3}\;\log(\sqrt{\kappa_{3}\kappa_{4}})\\ -\cos\beta_{t7}\;\sqrt{\frac{\kappa_{2}\sec^{2}\beta_{t7}}{\kappa_{3}+\kappa_{4}\tan^{2}\beta_{t7}}}(3\kappa_{1}^{2}\kappa_{3}\sqrt{\kappa_{3}+\kappa_{4}\tan^{2}\beta_{t7}}\;\log(\sqrt{\kappa_{4}\kappa_{3}+\kappa_{4}^{2}\tan^{2}\beta_{t7}}\\ +\kappa_{4}\;\tan\beta_{t7})+\kappa_{2}\sqrt{\kappa_{4}}\tan\beta_{t7})+3\kappa_{1}\kappa_{2}\sqrt{\kappa_{3}}\arctan(\frac{\sqrt{\kappa_{4}}\tan\beta_{t7}}{\sqrt{\kappa_{3}}})\}, \end{array}$$ (35)

where, the simplification parameters *κ*_1_, *κ*_2_, *κ*_3_, and *κ*_4_ can be expressed as,

$$\begin{array}{c} \kappa_{1}=r_{i}\cos\beta_{m},\\ \kappa_{2}=a_{o}^{2}b_{o}^{2}c_{o}^{2},\\ \kappa_{3}=c_{o}^{2}(a_{o}^{2}+b_{o}^{2}+\left(b_{o}^{2}-a_{o}^{2}\right)\cos(2(\theta_{o}-\phi_{m}))),\\ \kappa_{4}=2a_{o}^{2}b_{o}^{2}. \end{array}$$

Similarly, the marginal PDF of elevation AoA can be obtained by integrating Eq (33) over *ϕ*_*m*_ for appropriate limits,

$$\begin{array}{c} p\left(\beta_{m}\right)=\int_{-\pi }^{\pi }p\left(\phi_{m},\beta_{m}\right)d\phi_{m}. \end{array}$$ (36)

The marginal PDFs of elevation AoA given in Eq (36) can be obtained in closed-form; however, due to too large length of the closed-form equation, the solution is not presented here.

### 4.2 PDF of AoA at BS

The joint density function as a function of angles seen at the BS and the distance *r*_*b*_ can be found as,

$$p\left(r_{b},\phi_{b},\beta_{b}\right)=\frac{f(x_{b},y_{b},z_{b})}{\left|J(x_{b},y_{b},z_{b})\right|}.$$ (37)

The joint density function can be written as,

$$p\left(r_{b},\phi_{b},\beta_{b}\right)=\frac{r_{b}^{2}\cos\beta_{b}}{V_{i}}.$$ (38)

Integrating the joint density function given in Eq (38) over *r*_*b*_ ranging from 0 to *r*_*e*_ gives,

$$p\left(\phi_{b},\beta_{b}\right)=\frac{r_{e}^{3}\cos\beta_{b}}{3V_{i}},$$ (39)

where, *r*_*e*_ is the effective amount of scattering objects, which has different definition for different angular partitions of SR. *r*_*e*_ in a particular direction of observation (i.e., *ϕ*_*b*_ and *β*_*b*_) is calculated according to the geometry of the SR, as given in Algorithm 1. For a given *β*_*b*_ numerical values of *r*_*e*_ can be calculated for all the possible partitions of defined SR. The marginal PDF of azimuth AoA seen at BS can thus be obtained by integrating Eq (39) over *β*_*b*_ for its appropriate limits as under,

$$\begin{array}{c} p\left(\phi_{b}\right)=\int_{\beta_{\text{min}}}^{\beta_{t1}}p\left(\phi_{b},\beta_{b}\right)d\beta_{b}+\int_{\beta_{t1}}^{\beta_{t2}}p\left(\phi_{b},\beta_{b}\right)d\beta_{b}+\int_{\beta_{t2}}^{\beta_{t3}}p\left(\phi_{b},\beta_{b}\right)d\beta_{b}\\ +\int_{\beta_{t3}}^{\beta_{t4}}p\left(\phi_{b},\beta_{b}\right)d\beta_{b}+\int_{\beta_{t4}}^{\beta_{t5}}p\left(\phi_{b},\beta_{b}\right)d\beta_{b}+\int_{\beta_{t5}}^{\beta_{t6}}p\left(\phi_{b},\beta_{b}\right)d\beta_{b}+\int_{\beta_{t6}}^{\beta_{\text{max}}}p\left(\phi_{b},\beta_{b}\right)d\beta_{b}. \end{array}$$ (40)

Limits given in Eq (40) are for a particular scenario shown in Fig 3. Similarly, the marginal PDF of elevation AoA can be obtained by integrating Eq (39) over *ϕ*_*b*_ for appropriate limits,

$$\begin{array}{c} p\left(\beta_{b}\right)=\int_{\phi_{t_{o}}^{-}}^{\phi_{t_{i}}^{-}}p\left(\phi_{b},\beta_{b}\right)d\phi_{b}+\int_{\phi_{t_{i}}^{-}}^{\phi_{t_{i}}^{+}}p\left(\phi_{b},\beta_{b}\right)d\phi_{b}+\int_{\phi_{t_{i}}^{+}}^{\phi_{t_{o}}^{+}}p\left(\phi_{b},\beta_{b}\right)d\phi_{b}. \end{array}$$ (41)

The marginal PDFs of azimuth and elevation AoA given in Eqs (40) and (41) can be obtained using numerical integration techniques.

## 5 Temporal Characteristics of the Radio Channel

In this section, analytical expressions for the temporal statistics of the proposed model are derived. Expressions for joint and marginal PDF of ToA and AoA are derived for both up- and down-links. Let *τ* be the propagation delay of a multipath signal reflected from a certain scattering point (*s*_*p*_), located in the illuminated 3-D SR, which can be expressed in terms of distance of the propagation path and the propagation velocity (*c*) of radio wave, as

$$\tau =\frac{r_{m}+r_{b}}{c}.$$ (42)

The delay for line of sight (LoS) path is *τ*_*o*_, and the delay of longest propagation path is *τ*_max_, which can be expressed as,

$$\tau_{o}=\frac{\sqrt{d^{2}+h_{b}^{2}}}{c},$$ (43)

$$\tau_{\text{max}}=\{\begin{array}{cc} \frac{a_{o}+\sqrt{a_{o}^{2}+d^{2}+h_{b}^{2}+2a_{o}d\cos\theta_{o}}}{c} & ;\;0\leq \theta_{o}\leq \pm \frac{\pi }{2}\\ \frac{a_{o}+\sqrt{a_{o}^{2}+d^{2}+h_{b}^{2}+2a_{o}d\cos\left(\pi -\theta_{o}\right)}}{c} & ;\;\pm \frac{\pi }{2}<\theta_{o}\leq \pm \pi \end{array}.$$ (44)

When observing from MS in a given particular direction, delay of shortest and longest propagation path, (corresponded from the scatterers at the boundary of inner cylinder and outer ellipsoid), is shown by *τ*_*m*,min_ and *τ*_*m*,max_, respectively. These direction dependant limits on path delay can be computed as,

$$\tau_{m,\text{min}}\left(\phi_{m},\beta_{m}\right)=\frac{r_{i}+\sqrt{d^{2}+{\left(r_{i}\cos\beta_{m}\right)}^{2}-2dr_{i}\cos\beta_{m}\cos\phi_{m}+{\left(h_{b}-r_{i}\sin\beta_{m}\right)}^{2}}}{c},$$ (45)

$$\tau_{m,\text{max}}\left(\phi_{m},\beta_{m}\right)=\frac{r_{o}+\sqrt{d^{2}+{\left(r_{o}\cos\beta_{m}\right)}^{2}-2dr_{o}\cos\beta_{m}\cos\phi_{m}+{\left(h_{b}-r_{o}\sin\beta_{m}\right)}^{2}}}{c}.$$ (46)

**Algorithm 1**: Algorithm to numerically compute effective quantity of scattering points (*r*_*e*_) for given *ϕ*_*b*_ and *β*_*b*_.

1: **Start**

2: **if** *β*_*t*3_ < *β*_*t*1_ **then**

3:  **if** *β*_min_ ≤ *β*_*b*_ < *β*_*t*1_ **then** $r_{e}=\rho_{\beta o}^{-}-\rho_{\beta o}^{+}$

4:  **else**

5:   **if** *β*_*t*3_ ≤ *β*_*b*_ < *β*_*t*1_ **then** $r_{e}=\rho_{g}-\rho_{\beta o}^{+}$

6:   **else**

7:    **if** *β*_*t*2_ < *β*_*t*4_ **then**

8:     **if** *β*_*t*5_ ≤ *β*_*b*_ < *β*_*t*6_ **then** $r_{e}=\rho_{g}-\rho_{\beta o}^{+}$

9:     **if** *β*_*t*4_ ≤ *β*_*b*_ < *β*_*t*5_ **then** $r_{e}=\rho_{\beta i}^{+}-\rho_{\beta o}^{+}$

10:     **if** *β*_*t*2_ ≤ *β*_*b*_ < *β*_*t*4_ **then** $r_{e}=(\rho_{g}-\rho_{\beta i}^{-})+(\rho_{\beta i}^{+}-\rho_{\beta o}^{+})$

11:     **if** *β*_*t*1_ ≤ *β*_*b*_ < *β*_*t*2_ **then** $r_{e}=\rho_{g}-\rho_{\beta i}^{-}$

12:    **else**

13:     **if** *β*_*t*2_ ≤ *β*_*b*_ < *β*_*t*5_ **then** $r_{e}=\rho_{\beta i}^{+}-\rho_{\beta o}^{+}$

14:     **if** *β*_*t*1_ ≤ *β*_*b*_ < *β*_*t*4_ **then** $r_{e}=\rho_{g}-\rho_{\beta i}^{-}$

15:     **if** *β*_*t*5_ ≤ *β*_*b*_ < *β*_*t*6_ **then** $r_{e}=\rho_{g}-\rho_{\beta o}^{+}$

16:     **if** *β*_*t*4_ ≤ *β*_*b*_ < *β*_*t*2_ **then** *r*_*e*_ = 0

17: **else**

18:  **if** *β*_min_ ≤ *β*_*b*_ < *β*_*t*1_ **then** $r_{e}=\rho_{\beta o}^{-}-\rho_{\beta i}^{-}$

19:  **else**

20:   **if** *β*_*t*2_ ≥ *β*_*t*3_ **then**

21:    **if** *β*_*t*1_ ≤ *β*_*b*_ < *β*_*t*3_ **then** $r_{e}=\rho_{\beta o}^{-}-\rho_{\beta i}^{-}$

22:    **else**

23:     **if** *β*_*t*2_ < *β*_*t*4_ **then**

24:      **if** *β*_*t*4_ ≤ *β*_*b*_ < *β*_*t*5_ **then** $r_{e}=\rho_{\beta i}^{+}-\rho_{\beta o}^{+}$

25:      **if** *β*_*t*2_ ≤ *β*_*b*_ < *β*_*t*4_ **then** $r_{e}=\rho_{\beta i}^{+}-\rho_{\beta o}^{+}$

26:      **if** *β*_*t*5_ ≤ *β*_*b*_ < *β*_*t*6_ **then** $r_{e}=\rho_{g}-\rho_{\beta o}^{+}$

27:      **if** *β*_*t*3_ ≤ *β*_*b*_ < *β*_*t*2_ **then** $r_{e}=\rho_{g}-\rho_{\beta i}^{-}$

28:     **else**

29:      **if** *β*_*t*4_ ≤ *β*_*b*_ < *β*_*t*2_ **then** *r*_*e*_ = 0

30:      **if** *β*_*t*5_ ≤ *β*_*b*_ < *β*_*t*6_ **then** $r_{e}=\rho_{g}-\rho_{\beta o}^{+}$

31:      **if** *β*_*t*2_ ≤ *β*_*b*_ < *β*_*t*5_ **then** $r_{e}=\rho_{\beta i}^{+}-\rho_{\beta o}^{+}$

32:      **if** *β*_*t*3_ ≤ *β*_*b*_ < *β*_*t*4_ **then** $r_{e}=\rho_{g}-\rho_{\beta i}^{-}$

33:   **else**

34:    **if** *β*_*t*4_ ≤ *β*_*b*_ < *β*_*t*5_ **then** $r_{e}=\rho_{\beta i}^{+}-\rho_{\beta o}^{+}$

35:    **if** *β*_*t*2_ ≤ *β*_*b*_ < *β*_*t*3_ **then** $r_{e}=(\rho_{\beta o}^{-}-\rho_{\beta i}^{-})+(\rho_{\beta i}^{+}-\rho_{\beta o}^{+})$

36:    **if** *β*_*t*5_ ≤ *β*_*b*_ < *β*_*t*6_ **then** $r_{e}=\rho_{g}-\rho_{\beta o}^{+}$

37:    **if** *β*_*t*3_ ≤ *β*_*b*_ < *β*_*t*4_ **then** $r_{e}=(\rho_{g}-\rho_{\beta i}^{-})+(\rho_{\beta i}^{+}-\rho_{\beta o}^{+})$

38:    **if** *β*_*t*1_ ≤ *β*_*b*_ < *β*_*t*2_ **then** $r_{e}=\rho_{\beta o}^{-}-\rho_{\beta i}^{-}$

39: **End**

Similarly, when observing from BS, direction dependant limits on path delay, *τ*_*b*,min_ and *τ*_*b*,max_ can be found as,

$$\tau_{b,\text{min}}\left(\phi_{b},\beta_{b}\right)=\frac{\rho_{\beta o}^{+}+\sqrt{d^{2}+{\left(\rho_{\beta o}^{+}\cos\beta_{b}\right)}^{2}-2d\rho_{\beta o}^{+}\cos\beta_{b}\cos\phi_{b}+{\left(h_{b}-\rho_{\beta o}^{+}\sin\beta_{b}\right)}^{2}}}{c},$$ (47)

$$\tau_{b,\text{max}}\left(\phi_{b},\beta_{b}\right)=\frac{\rho_{\beta o}^{-}+\sqrt{d^{2}+{\left(\rho_{\beta o}^{-}\cos\beta_{b}\right)}^{2}-2d\rho_{\beta o}^{-}\cos\beta_{b}\cos\phi_{b}+{\left(h_{b}-\rho_{\beta o}^{-}\sin\beta_{b}\right)}^{2}}}{c}.$$ (48)

The parameter *r*_*b*_ is the distance of a particular scatterer from BS which can be obtained, in correspondence with *r*_*m*_, *ϕ*_*m*_, and *β*_*m*_, in simplified form as,

$$r_{b}\left(r_{m},\phi_{m},\beta_{m}\right)=\sqrt{r_{m}^{2}+d^{2}+h_{b}^{2}-2r_{m}\left(d\cos\beta_{m}\cos\phi_{m}+h_{b}\sin\beta_{m}\right)}.$$ (49)

Substituting Eq (49) in Eq (42) and solving for *r*_*m*_, the equation for *r*_*m*_ as function of path delay and angles seen as MS can be rearranged as

$$r_{m}\left(\tau ,\phi_{m},\beta_{m}\right)=\frac{c^{2}\tau^{2}-d^{2}-h_{b}^{2}}{2(c\tau -d\cos\beta_{m}\cos\phi_{m}-h_{b}\sin\beta_{m})}.$$ (50)

Similarly, the distance *r*_*b*_ of the scatterer from BS can be found in correspondence with path delay and angles seen at BS, as

$$r_{b}\left(\tau ,\phi_{b},\beta_{b}\right)=\frac{c^{2}\tau^{2}-d^{2}-h_{b}^{2}}{2(c\tau -d\cos\beta_{b}\cos\phi_{b}-h_{b}\sin\beta_{b})}.$$ (51)

The joint density function for ToA and AoA observed at MS can be found as,

$$p\left(\tau ,\phi_{m},\beta_{m}\right)=\frac{p(r_{m},\phi_{m},\beta_{m})}{|J(r_{m},\phi_{m},\beta_{m})|},$$ (52)

where, the transformation relation between *τ* and *r*_*m*_ is given in Eq (50). The Jacobean transformation *J*(*r*_*m*_, *ϕ*_*m*_, *β*_*m*_) shown in Eq (52), can thus be found as,

$$J\left(r_{m},\phi_{m},\beta_{m}\right)={\left|\frac{\partial r_{m}}{\partial \tau }\right|}^{-1},$$ (53)

$$J\left(r_{m},\phi_{m},\beta_{m}\right)=\frac{2{\left(d\cos\beta_{m}\cos\phi_{m}-c\tau +h_{b}\sin\beta_{m}\right)}^{2}}{c(d^{2}+h_{b}^{2}+c^{2}\tau^{2}-2c\tau \left(d\cos\beta_{m}\cos\phi_{m}+h_{b}\sin\beta_{m}\right))}.$$ (54)

Substituting Eqs (54) and (32) in Eq (52), the joint density function for ToA / AoA can be re-written in simplified form as,

$$p\left(\tau ,\phi_{m},\beta_{m}\right)=\frac{c{\left(d^{2}+h_{b}^{2}-c^{2}\tau^{2}\right)}^{2}\left(d^{2}+h_{b}^{2}+c^{2}\tau^{2}-2c\tau \left(d\cos\beta_{m}\cos\phi_{m}+h_{b}\sin\beta_{m}\right)\right)\cos\beta_{m}}{8V_{i}{\left(d\cos\beta_{m}\cos\phi_{m}-c\tau +h_{b}\sin\beta_{m}\right)}^{4}}.$$ (55)

The joint PDF of ToA in azimuth and elevation planes seen at MS can be found by integrating Eq (55) over elevation and azimuth angles, respectively.

$$p\left(\tau ,\phi_{m}\right)=\int_{0}^{\beta_{t7}}p\left(\tau ,\phi_{m},\beta_{m}\right)d\beta_{m},\;\;\tau_{m,\text{min}}<\tau \leq \tau_{m,\text{max}}\;\text{and}\;-\pi <\phi_{m}\leq \pi ,$$ (56)

$$p\left(\tau ,\beta_{m}\right)=\int_{-\pi }^{\pi }p\left(\tau ,\phi_{m},\beta_{m}\right)d\phi_{m},\;\;\tau_{m,\text{min}}<\tau \leq \tau_{m,\text{max}}\;\text{and}\;0\leq \beta_{m}\leq \frac{\pi }{2}.$$ (57)

Due to geometric symmetry, the ToA density functions observed at either end of the communication links take same mathematical form. This is because the same transformation of coordinates holds for up- and down-link, except the limits of angular and delay parameters [10]. Therefore, the joint density function for ToA and AoA seen at the BS can be expressed as,

$$p\left(\tau ,\phi_{b},\beta_{b}\right)=\frac{c{\left(d^{2}+h_{b}^{2}-c^{2}\tau^{2}\right)}^{2}\left(d^{2}+h_{b}^{2}+c^{2}\tau^{2}-2c\tau \left(d\cos\beta_{b}\cos\phi_{b}+h_{b}\sin\beta_{b}\right)\right)\cos\beta_{b}}{8V_{i}{\left(d\cos\beta_{b}\cos\phi_{b}-c\tau +h_{b}\sin\beta_{b}\right)}^{4}},$$ (58)

$$p\left(\tau ,\phi_{b}\right)=\int_{\beta_{\text{min}}}^{\beta_{\text{max}}}p\left(\tau ,\phi_{b},\beta_{b}\right)d\beta_{b},\;\;\tau_{b,\text{min}}<\tau \leq \tau_{b,\text{max}}\;\text{and}\;\phi_{to}^{-}\leq \phi_{b}\leq \phi_{to}^{+}.$$ (59)

As the geometry of the SR is different for different angular ranges, therefore, the angular limits of elevation AoA must be applied according to Algorithm 1.

$$p\left(\tau ,\beta_{b}\right)=\int_{\phi_{to}^{-}}^{\phi_{to}^{+}}p\left(\tau ,\phi_{b},\beta_{b}\right)d\phi_{b},\;\;\tau_{b,\text{min}}<\tau \leq \tau_{b,\text{max}}\;\text{and}\;\beta_{\text{min}}\leq \beta_{b}\leq \beta_{\text{max}}.$$ (60)

Thus the marginal PDF of ToA can be found, by integrating any of the Eqs (56)–(60) over corresponding angles for their appropriate limits, e.g.,

$$p\left(\tau \right)=\int_{-\pi }^{\pi }\int_{0}^{\beta_{t7}}p\left(\tau ,\phi_{m},\beta_{m}\right)\;d\beta_{m}\;d\phi_{m},\;\;\tau_{m,\text{min}}<\tau \leq \tau_{m,\text{max}}$$ (61)

## 6 Results and Discussion

This section presents the obtained analytical results along with a thorough analysis on spatial and temporal characteristics of the proposed channel model.

### 6.1 PDF of AoA observed at MS

**Fig 4:**
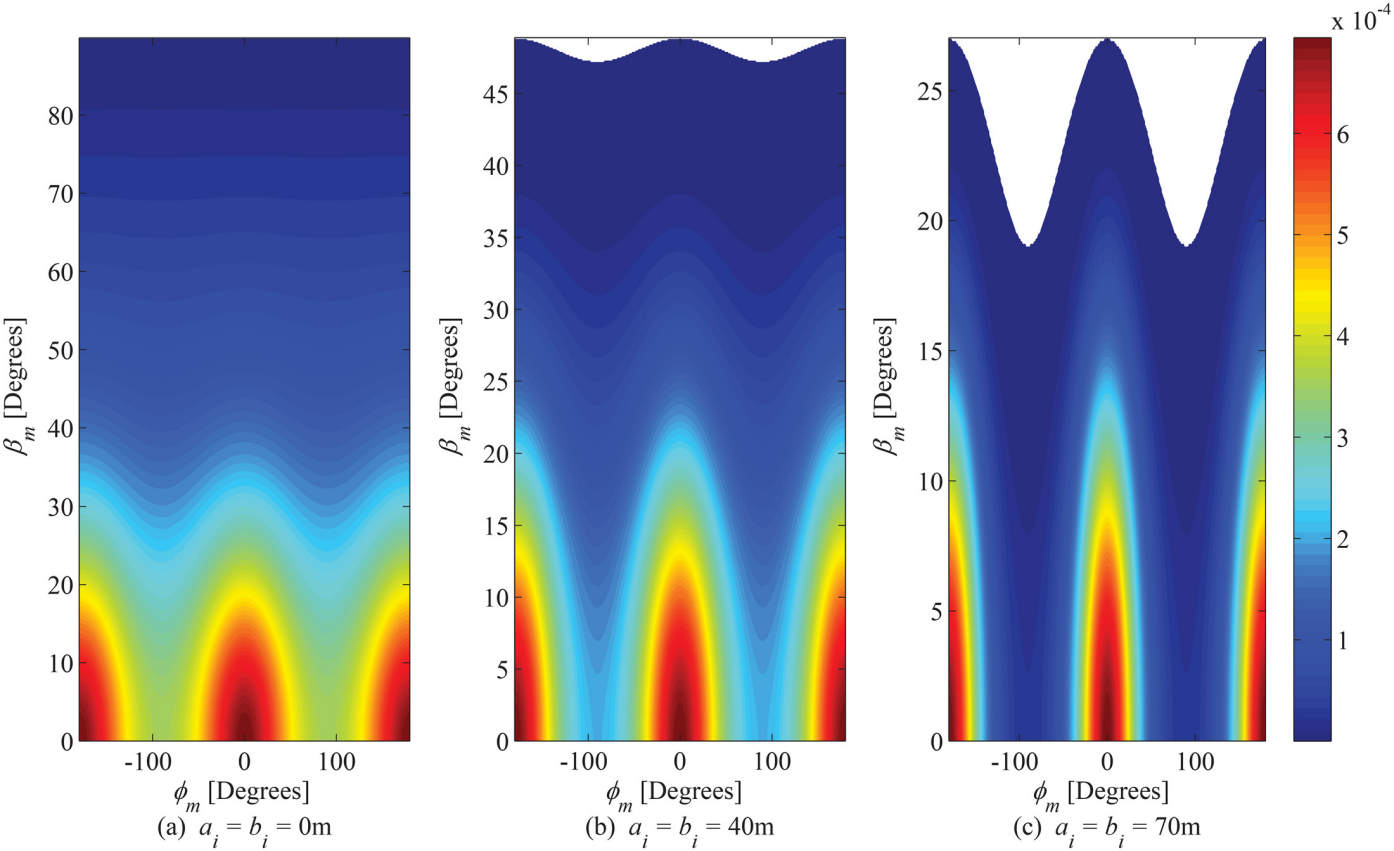
Effect of size of inner bounding elliptical-cylinder on joint PDF of AoA observed from MS. (*a*_*o*_ = 100m, *b*_*o*_ = 80m, *c*_*o*_ = 50m, and *θ*_*i*_ = *θ*_*o*_ = 0^*o*^).

**Fig 5:**
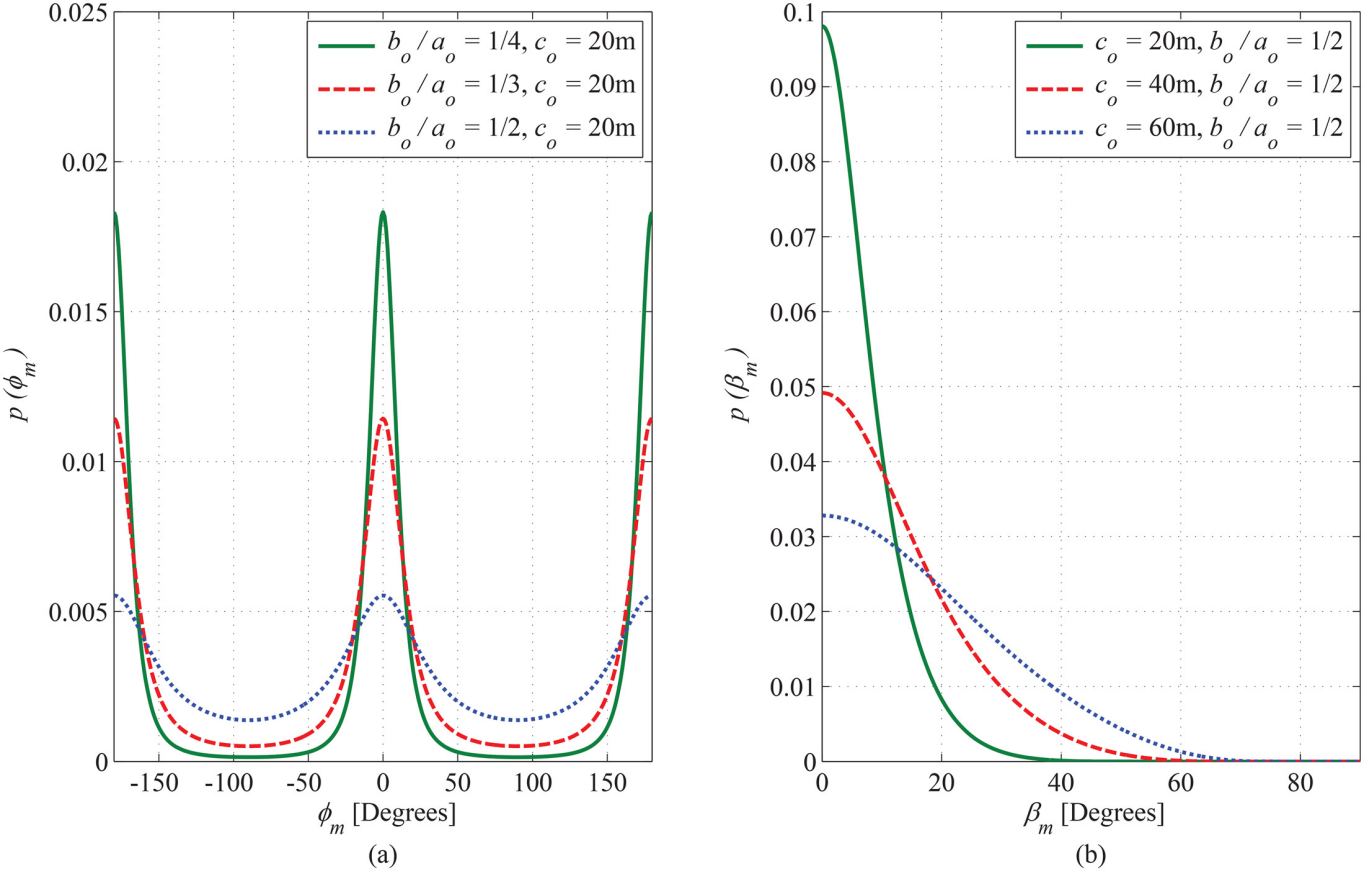
Effect of ratio of *b*_*o*_ to *a*_*o*_ on azimuth (a) and effect of *c*_*o*_ on elevation (b) Marginal PDFs of AoA observed from MS. (*a*_*o*_ = 100m, *a*_*i*_ = 30m, *b*_*i*_ = 15m, and *θ*_*i*_ = *θ*_*o*_ = 0^*o*^).

**Fig 6:**
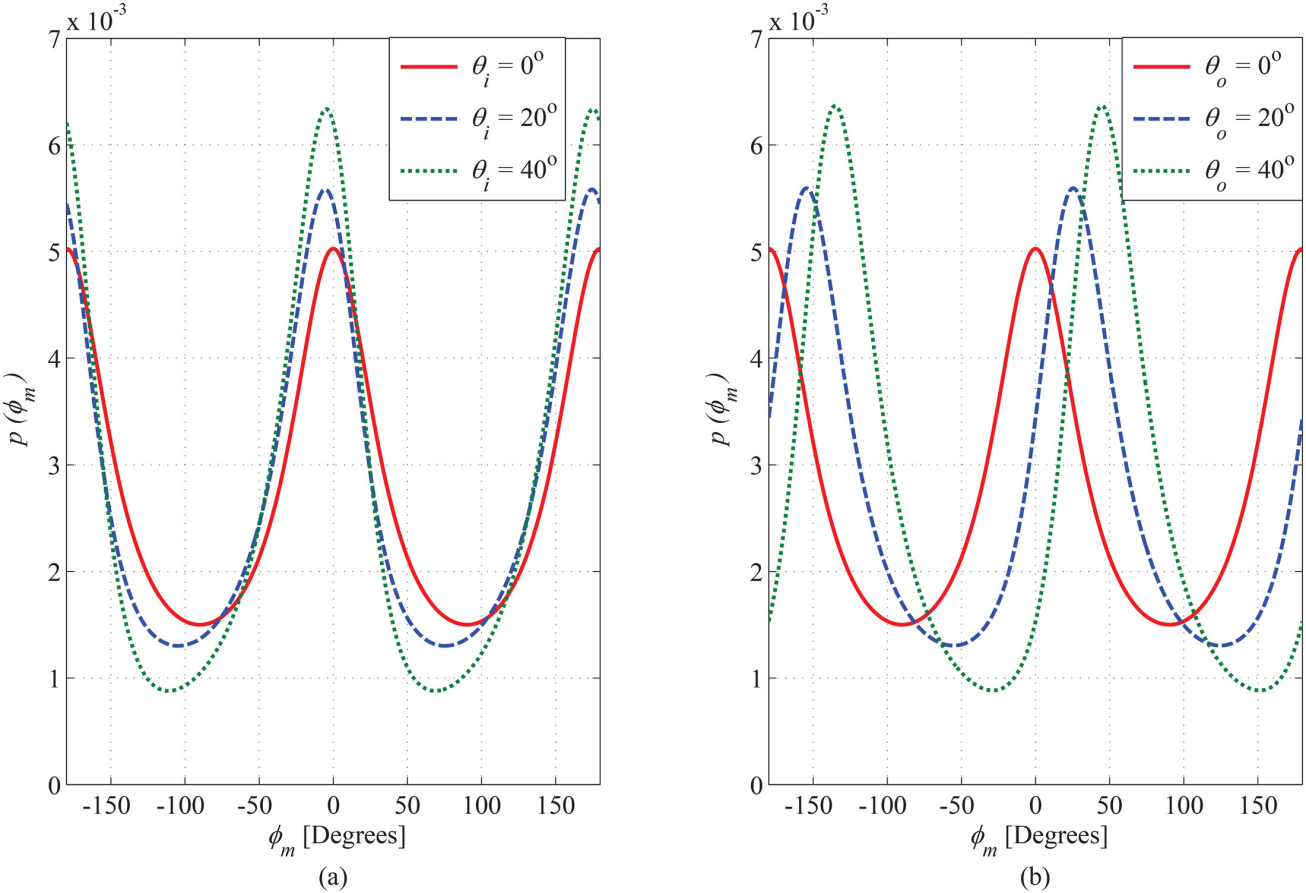
Effect of *θ*_*i*_ (a) and *θ*_*o*_ (b) on marginal PDFs of azimuth AoA observed from MS. (*a*_*o*_ = 100m, *b*_*o*_ = 60m, *c*_*o*_ = 50m, *a*_*i*_ = 30m, and *b*_*i*_ = 15m).

The joint PDF of AoA observed at MS is plotted in Fig 4, for different magnitudes of inner bounding elliptical-cylinder. It is evident that the introduction of inner cylinder decreases the angular span of elevation AoA. In order to elaborate the impact of the magnitude of SR in the azimuth plane on the PDF of azimuth AoA, the plots in Fig 5(a) are shown for different ratios between *a*_*o*_ and *b*_*o*_ by keeping the magnitude of hollow cylindrical region constant. It can be observed that the PDF approaches to uniform distribution, (the proposed model deduces to the Clarke’s model [26]), as the ratio between *a*_*o*_ and *b*_*o*_ approaches to 1. Furthermore, in order to show the impact of scattering objects in the elevation plane on the PDF of elevation AoA, the plots in Fig 5(b) are shown for different values of *c*_*o*_. It can be observed that the angular spread in elevation plane significantly increases with an increase in *c*_*o*_. This indicates the importance of inclusion of scatterers in elevation plane into the account for modeling the SR. The SR in the proposed model is designed as rotatable in horizontal plane around the MS to adapt various orientations of practical propagation environments; therefore, the impact of rotation of inner and outer bounding region on the PDF of azimuth AoA observing from MS has been plotted in Fig 6. It can be seen that the maximum value of PDF along LoS direction steers in azimuth axis with a certain angle by changing rotation parameters *θ*_*i*_ and *θ*_*o*_.

### 6.2 PDF of AoA Observed at BS

**Fig 7:**
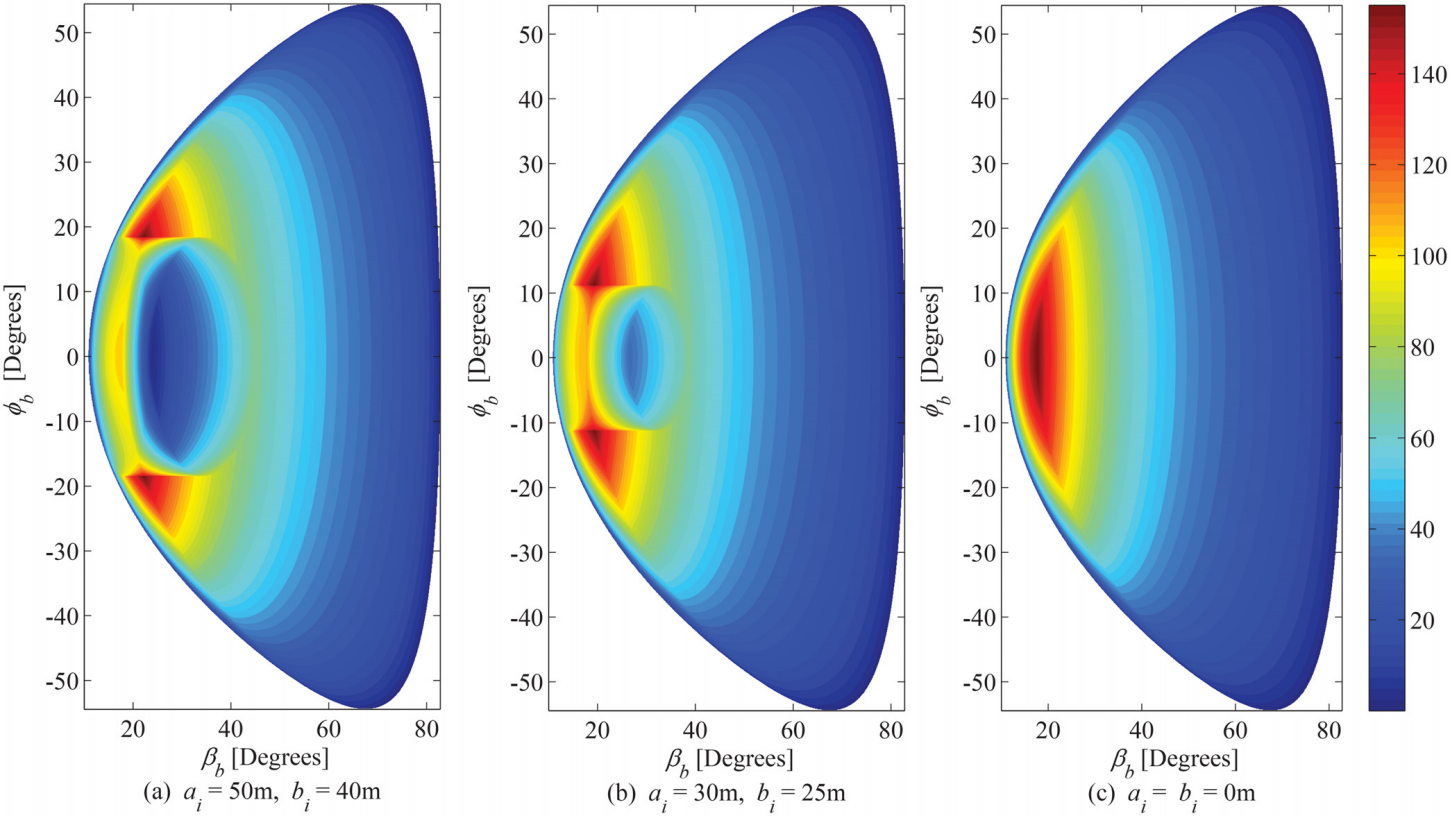
Joint PDF of AoA observed from BS. (*a*_*o*_ = 120m, *b*_*o*_ = 70m, *c*_*o*_ = 50m, *θ*_*i*_ = *θ*_*o*_ = 0^*o*^, *h*_*b*_ = 80m, and *d* = 130m).

**Fig 8:**
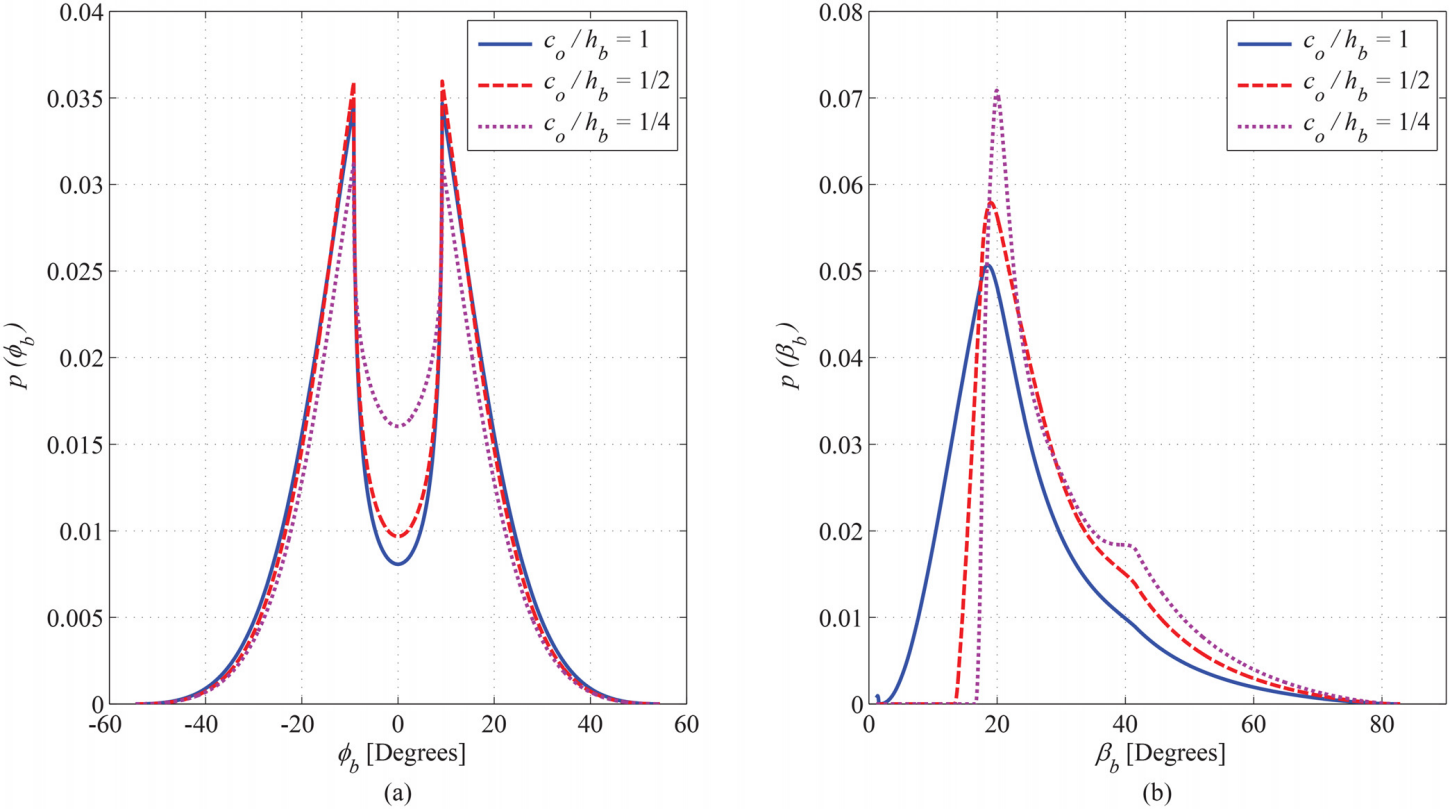
Effect of ratio of *c*_*o*_ to *h*_*b*_ on azimuth and elevation marginal PDF of AoA observed from BS. (*a*_*o*_ = 120m, *b*_*o*_ = 70m, *a*_*i*_ = 40m, *b*_*i*_ = 20m, *θ*_*i*_ = *θ*_*o*_ = 0^*o*^, and *d* = 130m).

**Fig 9:**
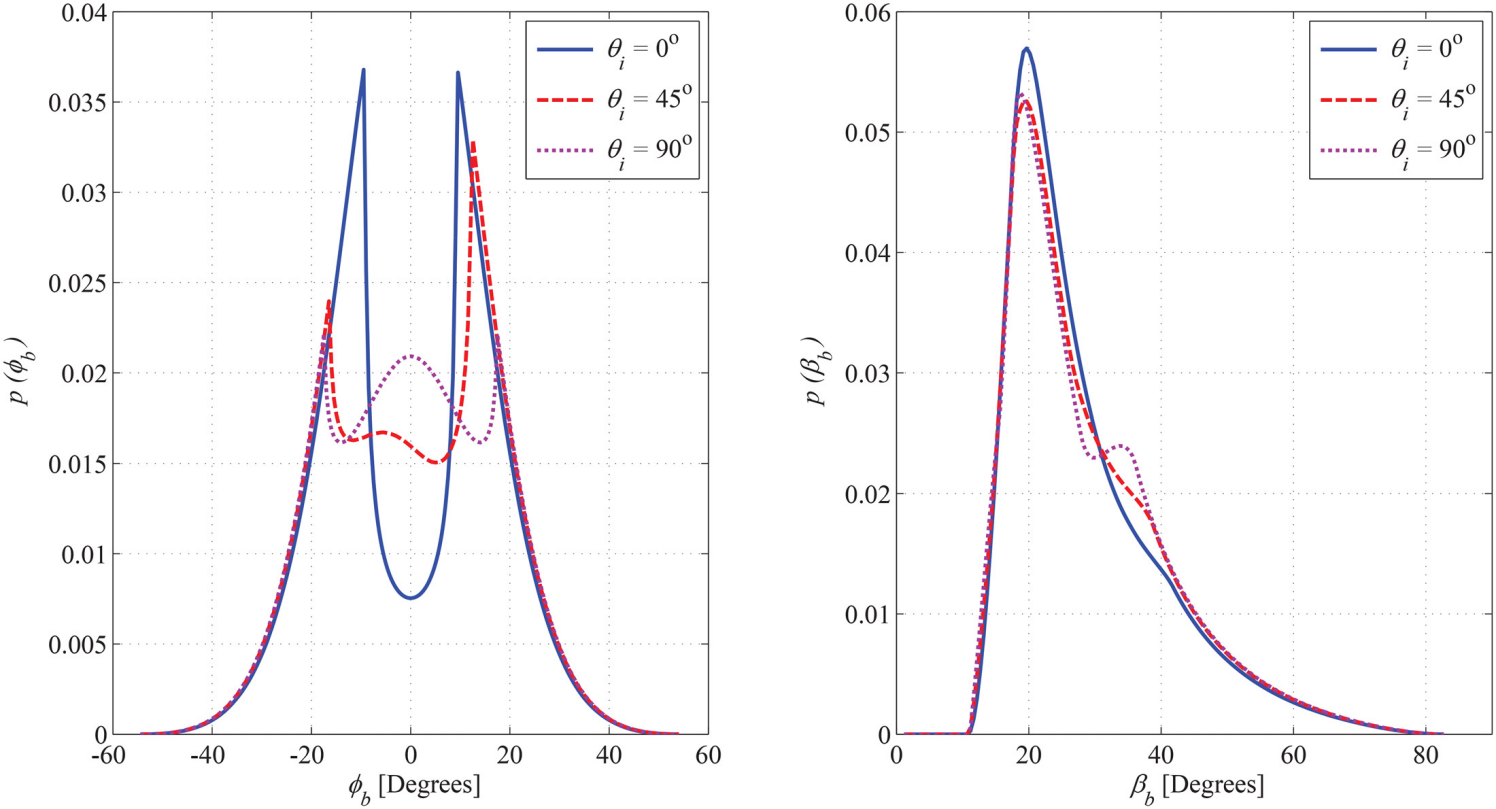
Effect of *θ*_*i*_ on azimuth and elevation marginal PDF of AoA observed from BS. (*a*_*o*_ = 120m, *b*_*o*_ = 70m, *c*_*o*_ = 50m, *a*_*i*_ = 40m, *b*_*i*_ = 20m, *θ*_*o*_ = 0^*o*^, *h*_*b*_ = 80m, and *d* = 130m).

The joint PDF of AoA in correspondence with azimuth and elevation angles observed at BS is plotted in Fig 7, for different values taken for the magnitude of the hollow region (i.e. *a*_*i*_ and *b*_*i*_). In future land mobile radio cellular communication networks, the cell size and the elevation of BS is expected to decrease [4]. In order to gauge the impact of elevation of the BS on the angular characteristics of the communication channel, the marginal PDF of AoA in azimuth and elevation planes is shown in Fig 8(a) and 8(b), respectively. Reducing the elevation of BS leads to a significant increase in the elevational angular spread, which accentuates the significance of considering the elevation plane for appropriate modeling. Effect of rotation of hollow elliptical-cylindric region on azimuth and elevation marginal PDFs of AoA seen at BS is shown in Fig 9(a) and 9(b), respectively.

### 6.3 PDF of ToA

**Fig 10:**
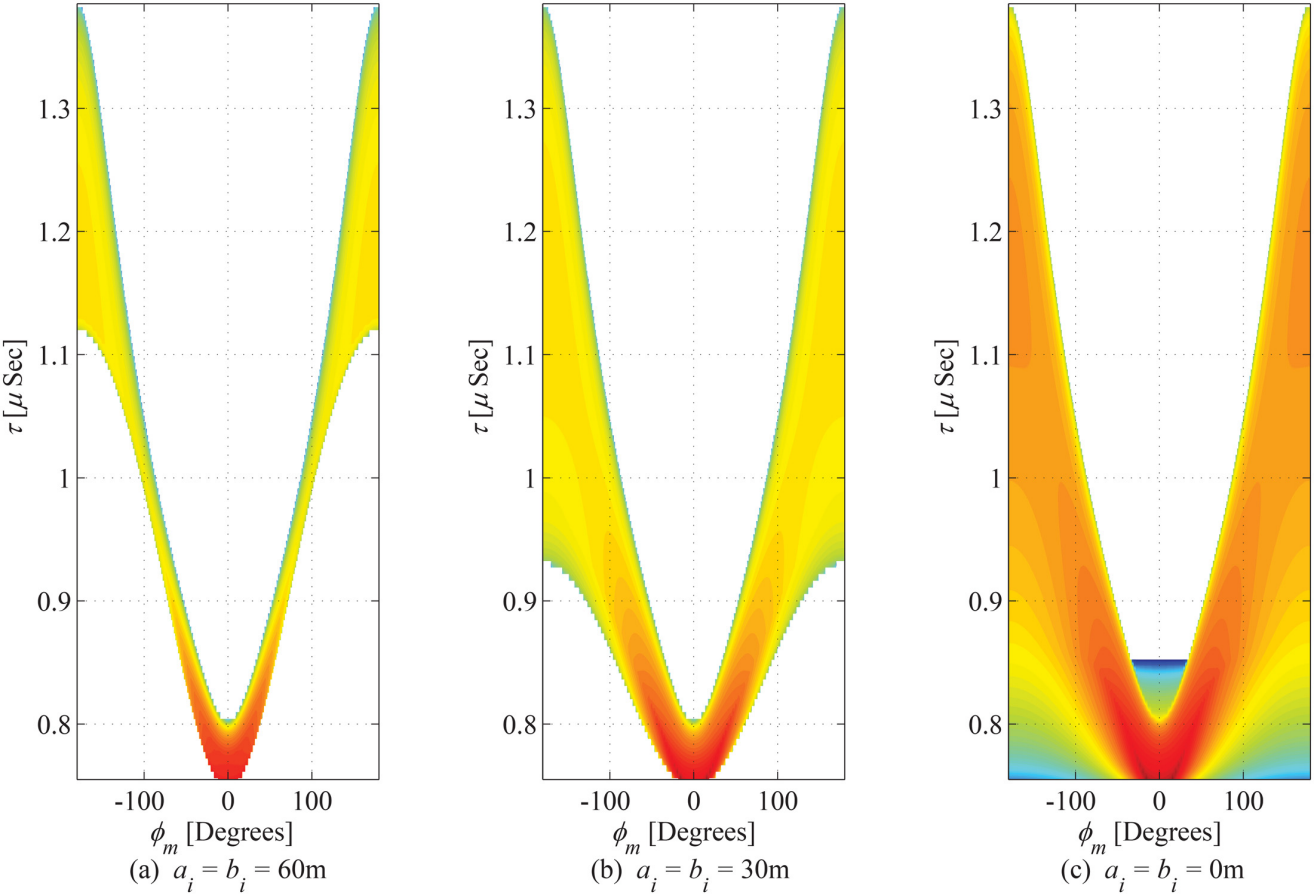
Joint PDF of ToA and azimuth AoA observed from MS. (*a*_*o*_ = 100m, *b*_*o*_ = 70m, *c*_*o*_ = 50m, *θ*_*i*_ = *θ*_*o*_ = 0^*o*^, *h*_*b*_ = 100m, and *d* = 200m).

**Fig 11:**
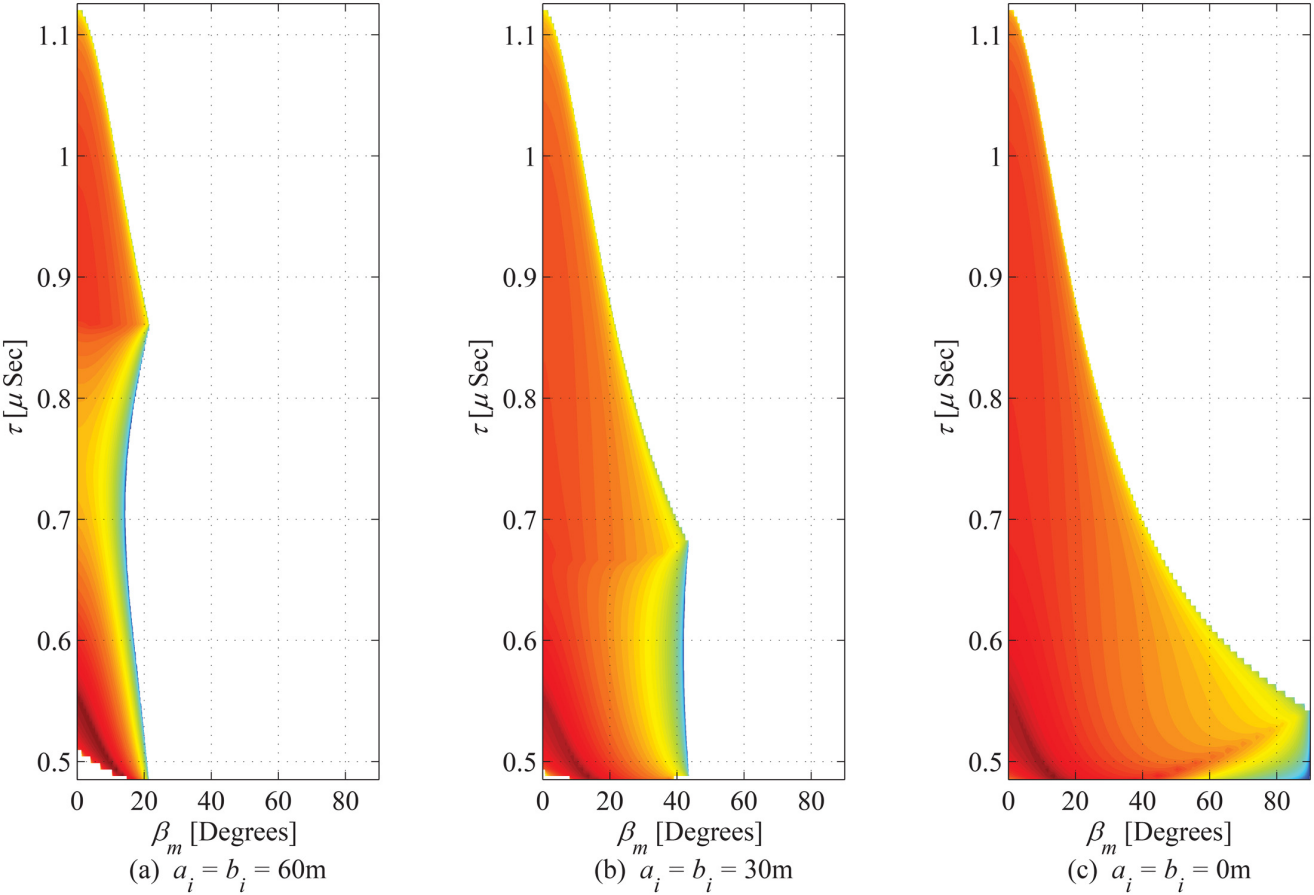
Joint PDF of ToA and elevation observed from MS. (*a*_*o*_ = 100m, *b*_*o*_ = 70m, *c*_*o*_ = 30m, *θ*_*i*_ = *θ*_*o*_ = 0^*o*^, *h*_*b*_ = 60m, and *d* = 130m).

**Fig 12:**
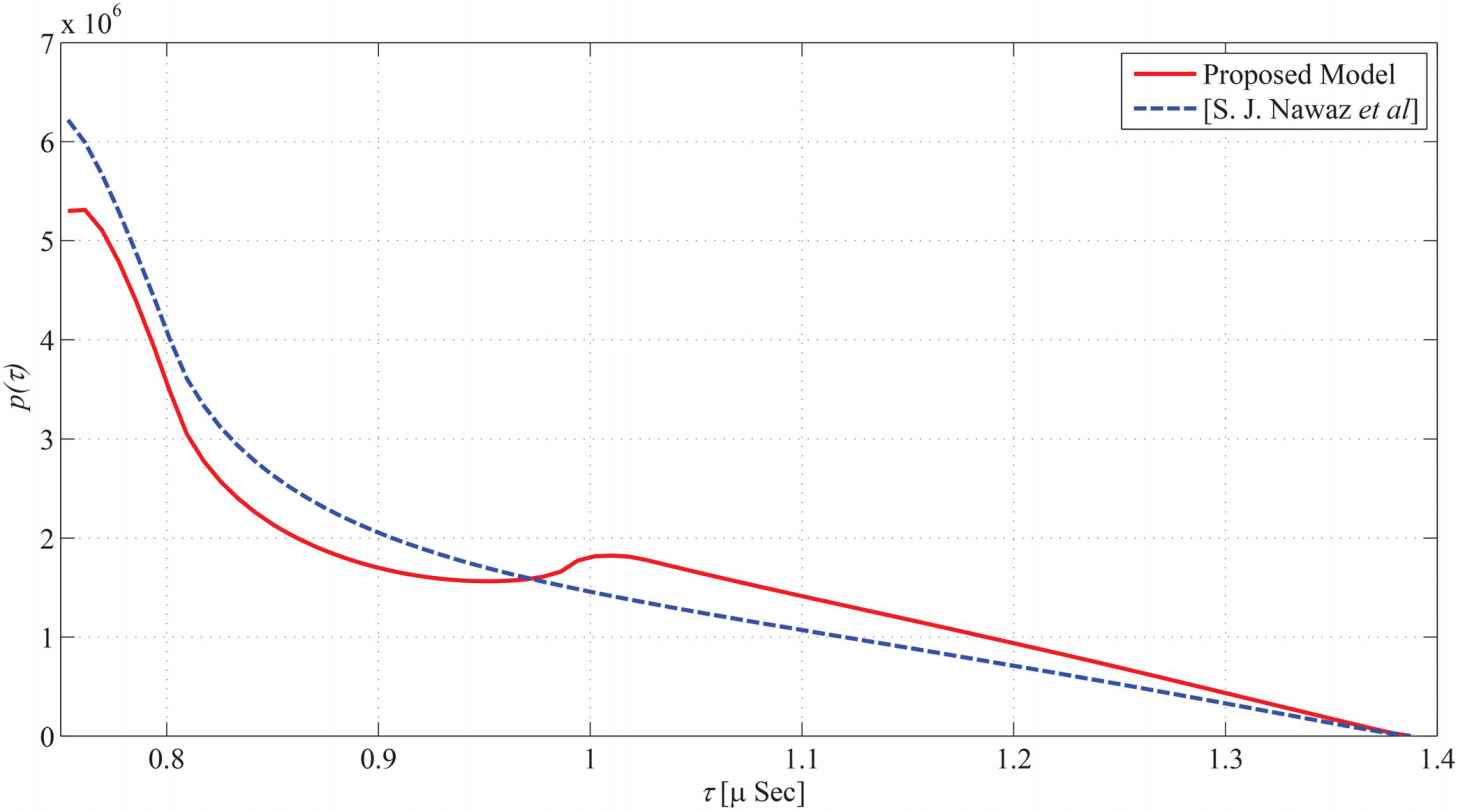
Marginal PDF of ToA of Proposed Model, and S. J. Nawaz. *et al* [11].

The joint PDF of ToA and azimuth AoA observed from MS is plotted in Fig 10(a), 10(b), and 10(c) for *a*_*i*_ and *b*_*i*_ set as 60m, 30m, and 0m, respectively. The increase in magnitude of hollow region results in increase in value of *τ*_*m*,min_ (i.e., deviation from *τ*_*o*_), which effectively changes the trend of distribution along the angles other than LoS direction. Joint PDF of ToA as seen from the MS is shown in Fig 11 and impact of inner hollow cylinder is studied. It is evident from the figure that by setting the close vicinity of the MS as scattering free region, (i.e., *a*_*i*_ > 0m, and *b*_*i*_ > 0m), the angular span of elevation AoA decreases; which further leads to a decrease in the delay spead. The marginal PDF of ToA seen from MS is shown in Fig 12. By a few appropriate substitutions for the parameters of the proposed model, the results deduce to those proposed in [11].

### 6.4 Validation of the proposed analytical model

In order to demonstrate the validity and generalization of the proposed 3-D model, a comparison of obtained theoretical results with various empirical data sets, analytical results of notable geometric channel models, and performed simulation results is presented in this section.

#### Data extraction and calibration

The discrete sample points of the data-sets provided in [27–29] are obtained by using graphical interpolation techniques. The extracted empirical data-sets are normalized to give unity area under the curve. The normalized data-sets and proposed analytical curves are shifted along vertical axis to align the peak valued sample point at unity. The extracted angular data is shifted to align the LoS direction of the empirical data-sets with the proposed analytical results. The analytical results are generated for the same amount of data points as those extracted from the empirical data-sets.

#### Goodness-of-fit metric

Least Squares Error (LSE) between the proposed analytical results and the extracted empirical data-sets is used as goodness-of-fit metric for measuring the fitness of data mappings. The LSE is defined as,

$$\text{LSE}=\frac{1}{N}\sum_{n=1}^{N}{\left[y\left(\phi_{n}\right)-p\left(\phi_{n}-\Delta \phi \right)\right]}^{2},$$ (62)

where, *y*(*ϕ*_*n*_) for *n* = 1… *N*, represents the normalized empirical data-set samples, *N* refers to the amount of data points, *p*(.) denotes the proposed analytical PDF of AoA, and Δ*ϕ* is a nuisance parameter to align the transmitter-receiver LoS AoA. The calibration searches for all the values of Δ*ϕ* to identify LSE.

#### Comparative Analysis

**Fig 13:**
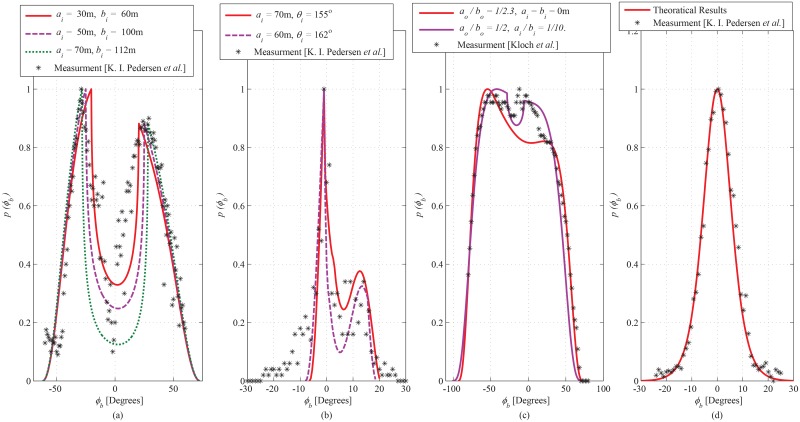
Curve-fitting proposed theoretical results to the empirical data in Pedersen [27] (Figure 14), Pedersen [28] (Figure 5), Kloch [29] (Figure 6), and Pedersen [27] (Figure 10), shown in (a), (b), (c) and (d), respectively.

**Fig 14:**
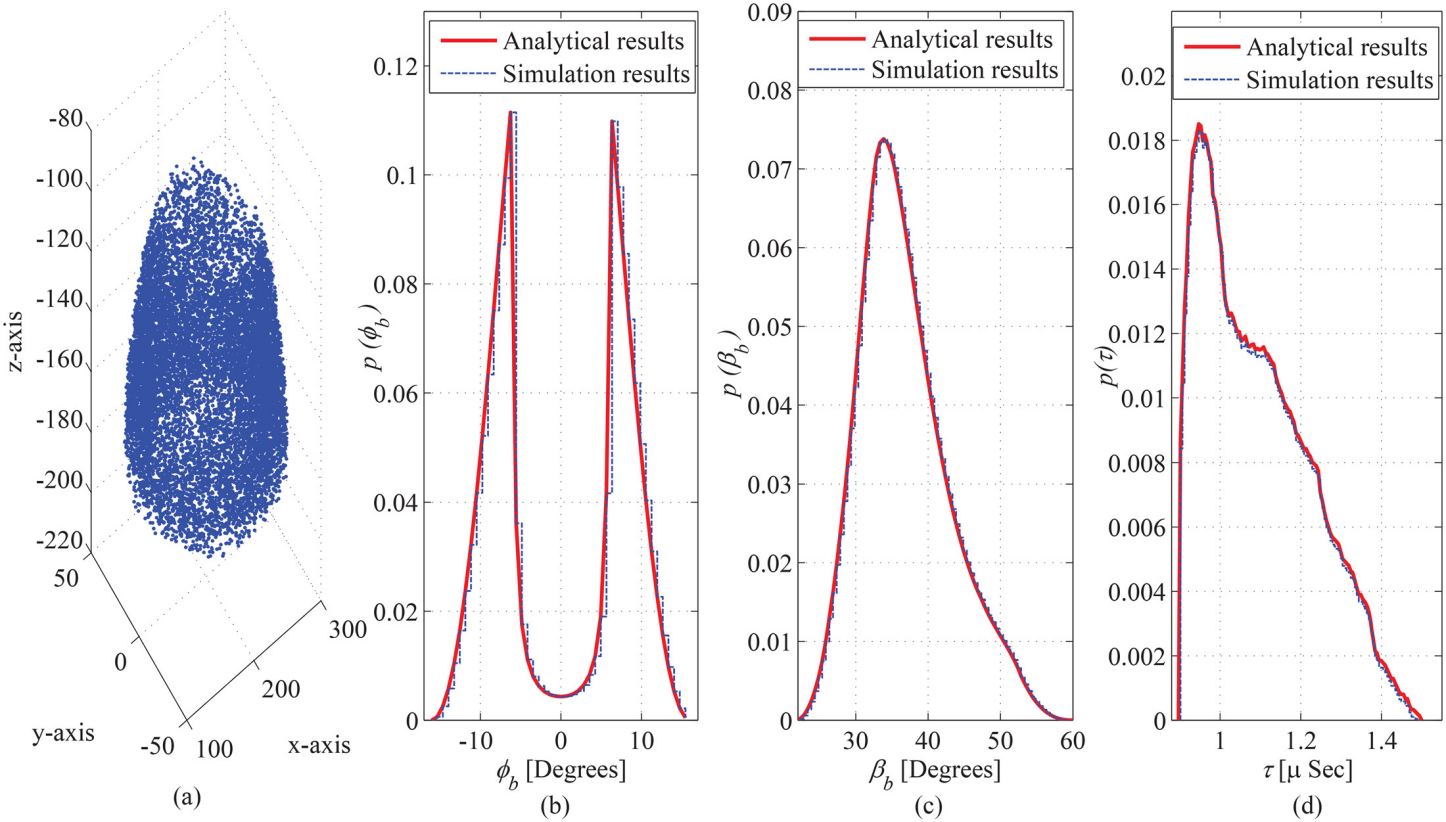
Simulation results, (a) spatial distribution of scattering objects used for simulation, comparison of analytical results with simulation results for marginal PDF of (b) azimuth AoA, (c) elevation AoA, and (d) ToA, observed from BS. of ToA.

**Fig 15:**
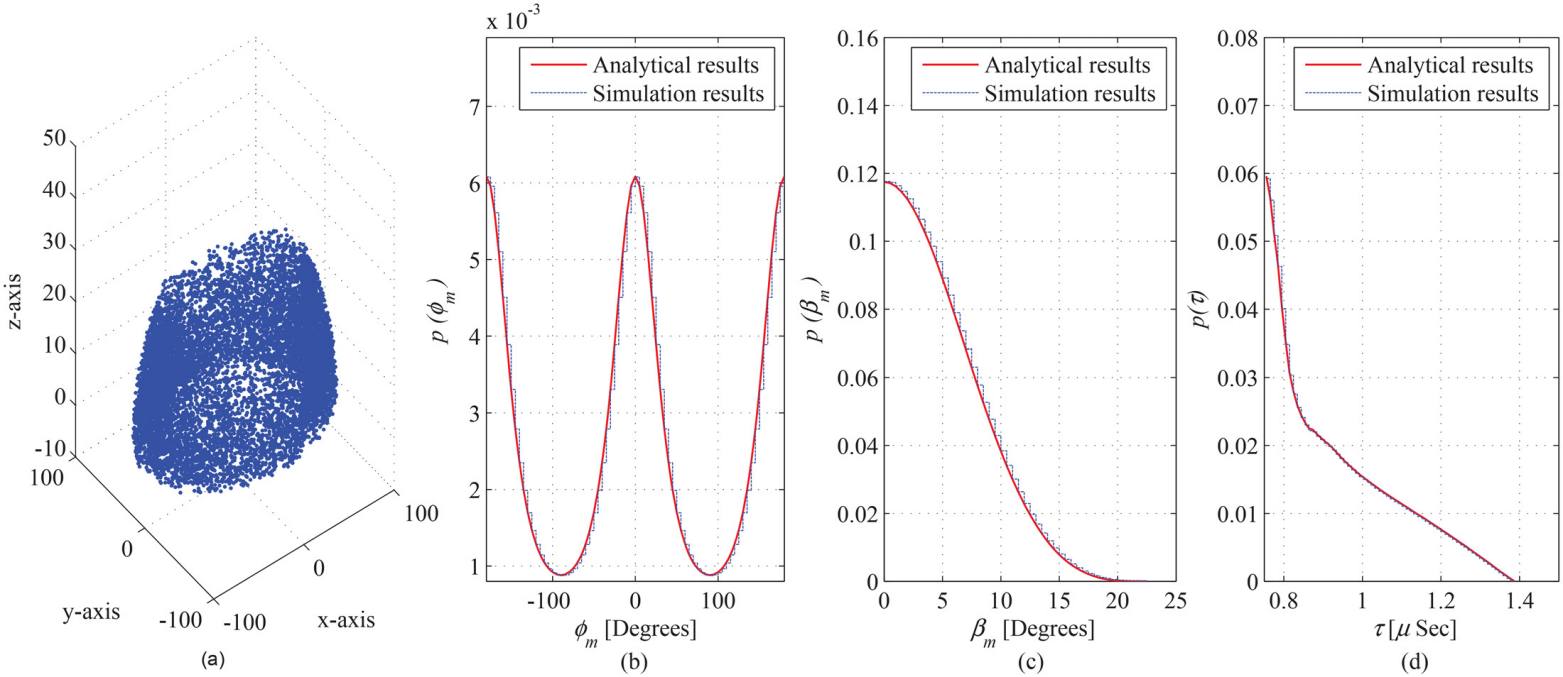
Simulation results, (a) spatial distribution of scattering objects used for simulation, comparison of analytical results with simulation results for marginal PDF of (b) azimuth AoA, (c) elevation AoA, and (d) ToA, observed from MS. of ToA.

A comparison of the obtained theoretical results with empirical data sets and performed simulation results is presented to demonstrate the validity of the proposed model. The obtained theoretical results for azimuth AoA observed at the BS are compared with the empirical data-sets obtained for different outdoor propagation environments given in Pedersen ([27], Figure 14), Pedersen ([28], Figure 5), Kloch ([29], Figure 6), and Pedersen ([27], Figure 10), shown in Fig 13(a), 13(b), 13(c) and 13(d), respectively. The geometric parameter substitutions taken for this comparison are shown in Table 2, where, the observed goodness-of-fit (i.e., LSE) is also demonstrated. The LSE of the compared analytical results is observed to be 0.0015, 0.0196, 0.022, and 0.0646 for the empirical data-sets in ([27], Figure 10), ([27], Figure 14), Pedersen ([28], Figure 5), and Kloch ([29], Figure 6), respectively. This good fit of proposed analytical results on the empirical data-sets establishes the validity of the proposed analytical results for AoA seen at BS. Due to unavailability (for the proposed particular scenario) of the empirical data sets for ToA and elevation AoA, the obtained analytical results for ToA and elevation AoA seen at BS and MS are validated through comparison with performed simulation results, as shown in Figs 14 and 15. In the performed computer simulations, the scattering points are generated in the defined SR, taken from uniform distribution, as shown in Figs 14(a) and 15(a). The simulated results for marginal PDFs of azimuth AoA, elevation AoA, and ToA observed at BS are compared with the proposed analytical results in Fig 14(b), 14(c), and 14(d), respectively. Similarly, the simulation and analytical results for spatiotemporal statistics seen at MS are shown in Fig 15(b), 15(c), and 15(d). For 10^7^ uniformly distributed scattering points, a good match is observed between simulation and analytical results, which establishes the validity of derived analytical expressions.

**Table 2:** Comparison of the proposed analytical model with some notable geometric models and empirical data sets found in the literature.

Geometric/ Empirical.	Models.	Environment Classification.	Elevation of BS.	Scattering Environment.	Parameters substituted for deducing to other models.	Least-Squares Error (LSE)
Geometric	Proposed.	Macrocell.	Elevated with *h*_*b*_.	Uniformly distributed scatterers confined in 3-D cylindrically hollowed ellipsoid.	-	-
	Olenko *et al.*[21]	Not specified.	Ground level.	Uniformly distributed scatterers confined in 2-D Hollow-Disk.	*a*_*o*_ = *b*_*o*_, *a*_*i*_ = *b*_*i*_, *c*_*o*_ = *h*_*b*_ = 0m.	-
	Zhou *et al.*[25]	Macrocell./ Microcell./ Picocell.	Ground level.	Uniformly distributed scatterers confined in 2-D Hollow-Disk.	*a*_*o*_ = *b*_*o*_, *a*_*i*_ = *b*_*i*_, *c*_*o*_ = *h*_*b*_ = 0m.	-
	Janaswamy. [9]	Macrocell.	Elevated.	Uniformly distributed scatterers confined in 3-D semi-spheroid (i.e., different major and minor axes).	*a*_*i*_ = *b*_*i*_ = 0m.	-
	Olenko *et al.*[10]	Not specified.	Ground level.	Uniformly distributed scatterers confined in 3-D hemispheroid (i.e., same major and minor axes).	*a*_*o*_ = *b*_*o*_, *c*_*o*_ = *a*_*i*_ = *b*_*i*_ = *h*_*b*_ = 0m.	-
	Baltzis *et al.*[14]	Macrocell.	Elevated.	Uniformly distributed scatterers confined in 2-D elliptical disc.	*c*_*o*_ = *a*_*i*_ = *b*_*i*_ = 0m.	-
Empirical	Pedersen *et al.*[27] (Figure 10)	Macrocell.	≫ Rooftop level.	Downtown, Aarhus, Denmark.	*a*_*o*_/*b*_*o*_/*c*_*o*_ = 9/2/7, *a*_*i*_ = *b*_*i*_ = 0m, *h*_*b*_/*d* = 1, *θ*_*i*_ = *θ*_*o*_ = 0^*o*^.	0.0015
	Pedersen *et al.*[27] (Figure 14)	Microcell.	Rooftop level (21m).	Riverside, Stockholm, Sweden.	*a*_*o*_/*b*_*o*_/*c*_*o*_ = 9/15/4, *a*_*i*_/*b*_*i*_ = 1/2, *h*_*b*_/*d* = 1/2.2, *θ*_*i*_ = 0^*o*^, *θ*_*o*_ = 10^*o*^.	0.0196
	Pedersen *et al.*[28] (Figure 5)	Microcell.	Rooftop level.	Downtown, Aarhus, Denmark.	*a*_*o*_/*b*_*o*_/*c*_*o*_ = 4/2/5, *a*_*i*_/*b*_*i*_ = 4/1, *h*_*b*_/*d* = 10/9, *θ*_*i*_ = 162^*o*^, *θ*_*o*_ = 0^*o*^.	0.022
	Kloch *et al.*[29] (Figure 6)	Outdoor (small campus).	Low (≈ 4m).	Campus, Aalborg University.	*a*_*o*_/*b*_*o*_/*c*_*o*_ = 10/20/7, *a*_*i*_/*b*_*i*_ = 1/10, *h*_*b*_/*d* = 1, *θ*_*i*_ = 0^*o*^, *θ*_*o*_ = 3^*o*^.	0.0646

The proposed model can be deduced to various outdoor propagation scenarios by substituting a few appropriate values for the model’s parameters (see, Table 2). The list of parameter substitutions for the proposed model to reduce to a few notable models proposed in [9, 10, 14, 21, 25] is provided in Table 2.

The proposed analytical results model the behavior of wireless propagation channels by providing a spatial and temporal probabilistic relationship between scatterer, transmitter, and receiver. The results provided for the PDFs of ToA and AoA are necessary tools to understand the behaviour of a radio propagation environment. The provided closed form expressions can be used for prediction of AoA and ToA statistics, which can further be used in determining shape factors (e.g., angular spread, angular constriction, direction of maximum fading, etc) and second order statistics (e.g.,level crossing rate, average fade duration). Closed-form analytical expressions are established as a computationally less complex solution when compared to the simulation method. The proposed analytical results can be helpful in designing precise antenna beamwidths based on azimuthal and elevational angular spread in accordance with the spacing between adjacent elements in a phased antenna array. The proposed analytical results can also be used as a reference for experimental measurement campaigns.

## 7 Future Work

The proposed analytical model can be extended by taking into account the mobility of one or both of the communicating nodes to study time-variability of the radio channel. A study on establishing a suitable choice of spatial distribution of the scatterers (e.g., hyperbolic, Gaussian, Poison, and/or subregions based clustering approaches, etc.) is of immense importance for a particular propagation scenario and a particular frequency range for the emerging communication networks. Selectivity in the spatial distribution of scatterers with respect to frequency range may be studied in the future. Moreover, the proposed model may be extended for more realistic scenario of the emerging communications networks by considering a suitable antenna’s radiation pattern employed at the BS.

## 8 Conclusion

A novel stochastic geometry-based tunable 3-D channel model for future generation land mobile radio communication networks has been proposed. A 3-D SR confined within an independently scalable and rotatable ellipsoid is designed around the MS hollowed with a scalable and rotatable elliptical-cylindric region. The SR is designed as tunable to adapt various physical communication scenarios by setting the model’s parameters linked with the different factors of physical propagation environments. Analytical expressions for joint and marginal PDFs of ToA and AoA have been derived for both up- and down-links. The obtained analytical results for angular and temporal statistics of the channel have been presented along with a comprehensive analysis. To provide insight on the obtained results, impact of various physical model parameters on spatio-temporal statistics of the channel have been presented. In order to establish validity of the proposed model, the obtained analytical results have been compared with various experimental datasets of different outdoor propagation scenarios in the literature and with the performed simulation results. A good fit of proposed results for angular statistics of the channel with various empirical datasets has been observed. Moreover, the obtained analytical results for temporal statistics show a good fit with simulation results for 10^7^ scattering points. It has been established that for future communication environments, i.e., small sized cells and low elevated BS, it is essential to model the SR as 3D space when modeling the vicinity of communicating nodes.

## Supporting Information

S1 Appendix Flowchart of the simulations performed for the calculation of azimuth and elevation AoA and ToA observing from MS is presented in S1 Appendix. Fig A in S1 Appendix shows flowchart of the simulation procedure observing from MS.
(PDF)
