# Supplementary material for: A 3-D Propagation Model for Emerging Land Mobile Radio Cellular Environments
Source: PLoS One. 2015 Aug 25;10(8):e0132555. doi: 10.1371/journal.pone.0132555 (PMC4549116; doi:10.1371/journal.pone.0132555)
Supplement: S1 Appendix — Fig A in S1 Appendix shows flowchart of the simulation procedure observing from MS. (PDF) [file pone.0132555.s001.pdf]

Appendix

Flowchart of the simulations performed for the calculation of azimuth and elevation AoA and ToA observing from MS is presented in this appendix. Fig. A shows flowchart of the simulation procedure observing from MS. Predefined number of scatterers N and geometric parameters of outer bounding ellipsoid and inner bounding elliptic-cylinder are given as input. Three random numbers m_1, m_2, and m_3, which are uniformly distributed in the range [0,1], are generated in each cycle of the simulation. Using these numbers azimuth angle (ϕ'), elevation angle (β'), and radial distance from MS to the scatterer(r'_m) are calculated. To identify the exact position of the scatterer a set of transformations is performed to calculate coordinates of the scatterer. r'_m is then compared with computed radial distances r'_i and r'_o to check if the considered scatterer lies within the effective SR. If the scatterer lies within the effective SR, azimuth and elevation AoA and ToA are calculated and stored, otherwise, a new scattering object is generated and procedure is repeated. This cycle is repeated until N number of samples are stored. Finally AoA and ToA PDFs are numerically calculated using the stored samples. Simulations for the statistics at BS are performed by adapting similar procedure.

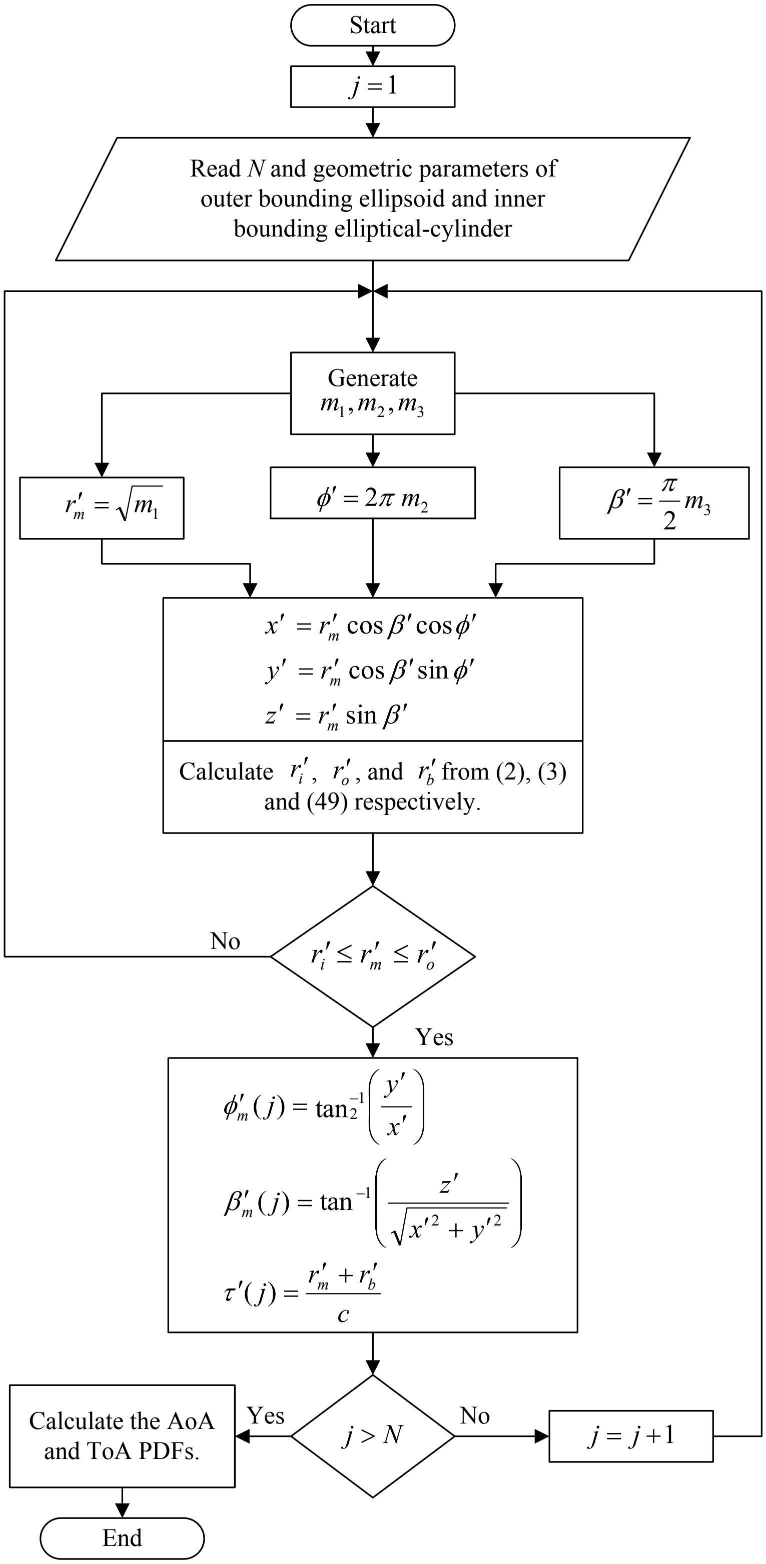

Fig. A. Flowchart of the Simulations Algorithm.
